# JNK1 Deficient Insulin-Producing Cells Are Protected against Interleukin-1*β*-Induced Apoptosis Associated with Abrogated Myc Expression

**DOI:** 10.1155/2016/1312705

**Published:** 2016-01-10

**Authors:** Michala Prause, Christopher Michael Mayer, Caroline Brorsson, Klaus Stensgaard Frederiksen, Nils Billestrup, Joachim Størling, Thomas Mandrup-Poulsen

**Affiliations:** ^1^Immuno-Endocrinology Lab, Endocrinology Research Section, Department of Biomedical Sciences, University of Copenhagen, 2200 Copenhagen N, Denmark; ^2^Section of Cellular and Metabolic Research, Department of Biomedical Sciences, University of Copenhagen, 2200 Copenhagen N, Denmark; ^3^Hagedorn Research Institute, Novo Nordisk, 2760 Måløv, Denmark; ^4^Copenhagen Diabetes Research Center, Herlev University Hospital, 2730 Herlev, Denmark; ^5^Biopharmaceuticals Research Unit, Novo Nordisk, 2760 Måløv, Denmark; ^6^Department of Molecular Medicine and Surgery, Karolinska Institutet, 17177 Stockholm, Sweden

## Abstract

The relative contributions of the JNK subtypes in inflammatory *β*-cell failure and apoptosis are unclear. The JNK protein family consists of JNK1, JNK2, and JNK3 subtypes, encompassing many different isoforms. INS-1 cells express JNK1*α*1, JNK1*α*2, JNK1*β*1, JNK1*β*2, JNK2*α*1, JNK2*α*2, JNK3*α*1, and JNK3*α*2 mRNA isoform transcripts translating into 46 and 54 kDa isoform JNK proteins. Utilizing Lentiviral mediated expression of shRNAs against JNK1, JNK2, or JNK3 in insulin-producing INS-1 cells, we investigated the role of individual JNK subtypes in IL-1*β*-induced *β*-cell apoptosis. JNK1 knockdown prevented IL-1*β*-induced INS-1 cell apoptosis associated with decreased 46 kDa isoform JNK protein phosphorylation and attenuated Myc expression. Transient knockdown of Myc also prevented IL-1*β*-induced apoptosis as well as caspase 3 cleavage. JNK2 shRNA potentiated IL-1*β*-induced apoptosis and caspase 3 cleavage, whereas JNK3 shRNA did not affect IL-1*β*-induced *β*-cell death compared to nonsense shRNA expressing INS-1 cells. In conclusion, JNK1 mediates INS-1 cell death associated with increased Myc expression. These findings underline the importance of differentiated targeting of JNK subtypes in the development of inflammatory *β*-cell failure and destruction.

## 1. Introduction

Inflammatory *β*-cell inhibition and apoptosis are increasingly accepted as key contributors to failing insulin secretion leading to type 1 and type 2 diabetes [1, 2]. Interleukin-1, a 17 kDa proinflammatory cytokine and mediator of inflammation, fever, and acute-phase responses [1, 3], inhibits *β*-cell function* in vitro* and* in vivo* by signaling via NF*κ*B and mitogen activated protein kinases (MAPK) to activate endoplasmic reticulum and mitochondrial death pathways [4–7]. The MAPK family encompasses the extracellular signal-regulated kinase (ERK), c-Jun N-terminal kinase (JNK), and p38 cascades. The precise contribution of the JNK pathway to stress-induced *β*-cell failure is not fully understood, especially with regard to the differential roles of the JNK subtypes, JNK1, JNK2, and JNK3.

The JNK proteins control the expression of immediate early genes such as AP-1 transcription factor family members and are important regulators of both apoptosis and survival, depending on the biological context [8, 9]. Three mammalian genes encoding for JNK have been identified:* jnk1*,* jnk2*, and* jnk3,* located on different chromosomes [10, 11]. JNK1 and JNK2 are expressed in a large variety of tissues regulating cell proliferation, differentiation, inflammation, autoimmunity, obesity, and tumorigenesis [12]. JNK3 expression was thought to be restricted to the brain, heart, and testes [13, 14]; however, more recently, JNK3 was found to be expressed also in human and mouse pancreatic *β*-cells [15]. All three JNK subtypes control cellular apoptosis. Mice deficient in a single JNK allele survive, as do* jnk1* ^−/−^ *jnk3* ^−/−^ and* jnk2* ^−/−^ *jnk3* ^−/−^ mice, whereas combined genetic disruption of* jnk1* and* jnk2* alleles causes early embryonic death due to severe dysregulation of apoptosis in the brain [14]. Additionally,* jnk1* ^−/−^ */jnk2* ^−/−^ mouse embryonic fibroblasts (MEFs) are protected against UV-induced apoptosis, indicating that JNK1 and JNK2 are required for a normal apoptotic response to UV exposure [16]. Furthermore,* jnk3* ^−/−^ mice are protected from stroke, neuronal death, and oxidative stress [17]. Supporting these direct observations of the importance of JNK in proapoptotic signalling, cell-permeable JNK inhibitory protein-1 (JIP-1) derived JNK inhibitory peptides or the JNK inhibitory small molecule SP600125 decrease intracellular JNK signalling and improve cell survival* in vitro* and* in vivo* [18–22].

Splicing of the* jnk* genes gives rise to more than twelve different transcript variants [13, 23] translating into proteins with and without a COOH-terminal extension to generate both 46 kDa and 54 kDa isoform proteins with a high level of homology [24]. Initially, the different JNK subtypes were thought to have largely redundant functions, but different tissue distribution, substrate preferences, and expression patterns support that the JNK subtypes also have nonredundant functions and are involved in distinct cellular processes [10, 12, 13, 23, 25]. However, little is known about how the individual JNK isoforms and subtypes mediate apoptosis. Apart from phosphorylating and activating members of the activating protein-1 (AP-1) transcription factor family, JNK proteins regulate other proteins involved in cell proliferation and apoptosis, including p53, Myc, and members of the Bcl-2 family of proteins [6, 26, 27].

The precise contribution of the individual JNK subtypes in mediating IL-1*β*-induced *β*-cell apoptosis is largely unknown, although a protective role of JNK3 in IL-1*β*, TNF-*α*, and IFN-*γ*-induced *β*-cell apoptosis via Akt2 signaling has been suggested [15, 28]. In these studies, no differentiation was made between the roles of the JNK subtypes in the signaling pathways of the three individual proinflammatory cytokines. Since JNK subtype activation is stimulus dependent and as IL-1*β* is indispensable in the proapoptotic combination of inflammatory cytokines, the action of TNF-*α* and INF-*γ* being mainly to synergize with IL-1*β*, it is important to investigate the differential role of the JNK subtypes in stimulus-specific pancreatic *β*-cell signalling.

Here, we investigated the individual roles of JNK1, JNK2, and JNK3 in IL-1*β*-induced apoptosis in insulin-producing INS-1 cells and provide evidence for a critical role of JNK1 in mediating IL-1*β*-induced *β*-cell apoptosis, whereas JNK2 but not JNK3 conferred protection.

## 2. Materials and Methods

### 2.1. Cell Culture and Reagents

The clonal rat *β*-cell line INS-1 [29] (gift from Wollheim, Geneva, Switzerland) and INS-1 cell lines stably expressing shRNA [30] were grown in RPMI-1640 medium with 11 mmol/L glucose (Invitrogen, Naerum, Denmark) supplemented with 50 *μ*mol/L *β*-mercaptoethanol, 100 U/mL penicillin, 100 *μ*g/mL streptomycin, and 10% heat-inactivated fetal bovine serum (FBS) (Invitrogen). Cells were incubated in a humidified atmosphere of 5% CO_2_ at 37°C. Recombinant mouse IL-1*β* was from BD Bioscience Pharmingen (San Diego, CA, USA).

JNK1(F-3) mouse monoclonal antibody raised against amino acid 1-384 of full length JNK1 p46 human origin was purchased from Santa Cruz Technologies (Santa Cruz, CA, USA, catalog number: sc-1648, used at 1 : 1000 dilution). JNK2 rabbit polyclonal antibody raised against a synthetic peptide for human JNK2 (catalog number: #4672, used at 1 : 1000 dilution), JNK3 rabbit monoclonal antibody raised against a synthetic peptide for human JNK3 (catalog number: #55A8, used at 1 : 1000 dilution), P-JNK (Thr183/Tyr185) polyclonal rabbit antibody raised against a synthetic phosphopeptide corresponding to residues surrounding Thr183/Tyr185 of human SAPK/JNK (catalog number: #9251, used at 1 : 1000 dilution), T-JNK polyclonal rabbit antibody raised against a recombinant human JNK2 fusion protein (catalog number: #9252, used at 1 : 1000 dilution), cleaved caspase 3 (Asp175) rabbit polyclonal antibody raised against amino terminal residues adjacent to Asp175 in human caspase 3 (catalog number: #9661, used at 1 : 500 dilution), Myc (D84C12) rabbit monoclonal antibody raised against synthetic peptide corresponding to amino-terminal residues of c-Myc (catalog number: #5605, used at 1 : 1000 dilution), and *β*-tubulin (9F3) rabbit monoclonal antibody raised against human *β*-tubulin (catalog number: #2128, used at 1 : 1000 dilution) were all from Cell Signalling (Beverly, MA, USA). The mouse monoclonal *β*-actin antibody raised against *β*-cytoplasmic actin N-terminal peptide, Ac-Asp-Asp-Asp-Ile-Ala-Ala-Leu-Val-Ile-Asp-Asn-Gly-Ser-Gly-Lys, conjugated to Keyhole Limpet Haemocyanin (KLH) was obtained from Abcam (Cambridge, UK, catalog number: ab6276, used at 1 : 10000 dilution). The specificity of the JNK antibodies was previously verified against recombinant JNK1, JNK2, and JNK3 protein using Western blot analysis [30].

HEK293FT cells, used to produce Lentivirus, were cultured in D-MEM medium (Invitrogen) supplemented with 100 U/mL penicillin, 10 nM MEM nonessential amino acids, 1 mM sodium pyruvate, and 10% FBS (Invitrogen) (complete D-MEM).

HT1080 cells, used for virus titration, were cultured in D-MEM medium (Invitrogen) supplemented with 100 U/mL penicillin, 100 *μ*g/mL streptomycin, and 10% FBS (Invitrogen).

#### 2.1.1. Lentivirus Expressing shRNA and INS-1 Stable Cell Line Production

INS-1 cell lines, stably expressing JNK1, JNK2, or JNK3 shRNAs, nonsense shRNA, or empty vector, were created as described in [31]. In brief, INS-1 cells were transduced with an MOI of 5 with virus generated by transfection of HEK293FT cells with the Lentiviral vectors pLKO.1 JNK1 (TRCN0000055115), pLKO.1 JNK2 (TRCN0000012590), pLKO.1 JNK3 (TRCN0000012634), or empty vector (all from Open Biosystems, Thermo Scientific, St. Leon-Rot, Germany) or pLKO.1 nonsense (SHC002) (Sigma, Brondby, Denmark). All constructs contained a puromycin-resistance gene as mammalian selection marker, and stably transduced INS-1 cells were selected using 1 *μ*g/mL puromycin (Sigma). INS-1 cell lines were passaged a minimum of three times and tested for knockdown efficiency and specificity compared to empty vector and nonsense shRNA expressing INS-1 cell lines.

### 2.2. Microarray Analysis

Total RNA was isolated using Trizol (Sigma) followed by RNeasy Mini Elute Kit (Qiagen, Hilden, Germany) purification. 1 *µ*g of total RNA was used to prepare targets by One-Cycle Target labeling kit (Affymetrix, Santa Clara, CA, USA) following the instructions of the manufacturer. Hybridization cocktails were hybridized onto Rat Genome 230 2.0 GeneChips, containing 31,099 rat gene probes, at 45°C for 17 h (60 Rpm) in a Hybridization Oven 640 (Affymetrix). GeneChips were rinsed and stained in a GeneChip fluidics station 450 using the fluidics protocol “EukGE-WS2v5_450” (Affymetrix). Chips were scanned in a GeneChip scanner 3000 (Affymetrix). For all conditions, three separate experiments were analyzed on separate arrays; that is, a total of 36 arrays were used. Array data is available at Arrayexpress [31] (https://www.ebi.ac.uk/arrayexpress), accession number E-MTAB-3146.

#### 2.2.1. Microarray Data Preprocessing and Visualization

To adjust for nonspecific hybridization, optical effects, and comparability, probe intensities were combined and normalized using the RMA method. All intensities were log_2_-transformed. Significant differential expression was assessed using the moderated *t*-statistics for pairwise comparison between conditions implemented in the LIMMA package. The comparisons performed were 45 min IL-1*β* exposed JNK1 knockdown (KD) INS-1 cells adjusted for nonexposed JNK1 KD versus 45 min IL-1*β* exposed NS control INS-1 cells adjusted for nonexposed NS control INS-1 cells; 45 min IL-1*β* exposed JNK2 KD INS-1 cells adjusted for nonexposed JNK2 KD versus 45 min IL-1*β* exposed NS control INS-1 cells adjusted for nonexposed NS control INS-1 cells; and 45 min IL-1*β* exposed JNK3 KD INS-1 cells adjusted for nonexposed JNK3 KD versus 45 min IL-1*β* exposed NS control INS-1 cells adjusted for nonexposed NS control INS-1 cells. Genes were considered to be significantly regulated if the log_2_ fold change was >1 or <−1 and the *P* value was <0.05. Only probes that could be mapped to gene identifiers using the “rat2302.db” probe annotation package were considered for further analysis.

For clustering analysis, we used hierarchical clustering as implemented in the heatmap.2 function in the gplots r package. Briefly, the mean log_2_ expression value for three replicates of the 12 conditions was calculated for each probe. Overrepresented biological processes among groups of regulated genes were identified by hypergeometric testing of gene ontology (GO) terms using DAVID [32, 33]. Only GO terms reaching a Benjamini corrected *P* value <0.05 were considered significantly overrepresented.

### 2.3. cDNA and qRT-PCR

INS-1 cells were exposed to 150 pg/mL IL-1*β* or vehicle over either a 12 h (stable shRNA cell line experiments) or 24 h (INS-1 cell line experiments) time course and total RNA was isolated using the RNeasy kit (Qiagen) and quantified on a NanoDrop 1000 microvolume spectrophotometer. The RNA was treated with recombinant shrimp DNase (Affymetrix), and first strand cDNA was synthesized from 2 *μ*g of RNA using the High Capacity cDNA Reverse Transcription Kit (Applied Biosystems, Carlsbad, CA, USA) following the manufacturer's protocol. Quantitative RT-PCR was performed in a 10 *μ*L volume using 0.5 *μ*L of premixed Taqman primer/probes (Applied Biosystems), 5 *μ*L 2x Taqman Universal Master Mix (Applied Biosystems), and 4.5 *μ*L template (20 ng) and run on a 384-well plate in triplicate on the Applied Biosystems Prism 7900HT real-time PCR machine for 40 cycles. The results were analyzed using SDS 2.4 software (Applied Biosystems). The following primer/probes were purchased from Applied Biosystems: Hprt1 (Rn01527840_m1), RN18S1 (Hs03928990_g1), JNK1 (Rn01453358_m1), JNK2 (Rn00569058_m1), JNK3 (Rn00563035_m1), Jun (Rn00572991_s1), Trp53 (Rn00755717_m1), Junb (Rn00572994_s1), Jund (Rn00824678_s1), and Myc (Rn00561507_m1). The primer/probe sequences for the JNK isoforms are listed in Table 1 and synthesized by Integrated DNA Technologies (IDT, Berchem, Belgium). We utilized the human isoform-specific primer and probe sequences determined by Dreskin et al. [34] and modifying them based upon a cross species homology comparison between human and mouse, as the rat JNK isoform sequences were not published. Relative transcript quantities were calculated by the standard curve method and normalized to the average of the housekeeping genes 18S and Hprt1. The appropriate housekeeping genes were selected by analyzing the mRNA expression of four common housekeeping genes, GAPDH, PPIA, 18S, and Hprt1, in INS-1 cells after 12 and 24 h exposures to IL-1*β*. The two genes used were the least variable following IL-1*β* treatment.

### 2.4. Transfection Studies

INS-1 cells (0.8 × 10^6^ cells/well for Western blotting and 0.075 × 10^6^ cells/well for Cell Death Detection Assay) were transfected with siRNA directed against rat Myc (Sigma), negative control siRNA (mission siRNA universal negative control, Sigma), or vehicle. The cells were transfected using DharmaFECT4 (Thermo Scientific, Denmark) according to the manufacturer's protocol, with a final concentration of siRNA of 50 nmol/L. The cells were incubated for 22 h with siRNA or vehicle and then exposed to IL-1*β* (150 pg/mL) or left nonexposed for 24 h. Protein was isolated and analyzed by Western blotting and apoptotic cell death was measured by the Cell Death Detection ELISA^PLUS^ (Roche, Hvidovre, Denmark).

### 2.5. Western Blot Analysis

INS-1 cells or INS-1 cell lines stably expressing shRNA were grown to 80–90% confluence, exposed to IL-1*β* (150 pg/mL) or vehicle for up to 24 h, washed in ice-cold PBS, and lysed for 15 min on ice using 1x lysis buffer (Cell Signaling). The protein concentration was measured by the Bradford method (Bio-Rad, Copenhagen, Denmark). 30–40 micrograms of total protein were mixed with 4x LDS sample buffer (Invitrogen), heated for 5 min at 70°C, and loaded on 10% NuPAGEBis-Tris gel (Invitrogen). Proteins were blotted onto nitrocellulose filter membranes (Invitrogen). Ponceau staining measuring total protein was used as loading control. The blots were blocked for 1 h in 1x tris-buffered saline (pH 7.6) containing 0.1% Tween 20 and 5% BSA and afterwards incubated overnight at 4°C with primary antibody. Blots were rinsed and incubated with a rabbit secondary horseradish peroxidase-conjugated antibody for 1 h. Immune complexes were detected by chemiluminescence using Super Signal West Dura Extended Duration Substrate (Thermo Scientific), and images were captured digitally by use of the Fuji LAS3000 platform (Fujifilm, Tokyo, Japan) or the Alpha Innotech FlourChem Q imaging platform (Kem-En-Tec, Taastrup, Denmark).

### 2.6. Cell Death Detection Assay

Apoptotic cell death was measured by the detection of DNA-histone complexes released from the nucleus to the cytosol of cells by using Cell Death Detection ELISA^PLUS^ (Roche) as described by the manufacturer. In brief, 0.075 × 10^6^ INS-1 cells stably expressing JNK1, JNK2, JNK3, nonsense shRNA, or empty vector were cultured 24 h prior to exposure to IL-1*β* (150 pg/mL) or vehicle. After an additional 24 h, the culture medium was removed and cells lysed in 200 *μ*L 1x lysis buffer for 30 min at room temperature. The lysate was then centrifuged for 10 min at 200 ×g, incubated with anti-DNA peroxidase and anti-histone-biotin, and added to streptavidin-coated wells for 2 h at room temperature. Absorbance was measured after addition of peroxidase substrate ABTS (2.2-azino-bis-3-ethylbenzthiazoline-6-sulfonate) at 405 and 490 nm.

### 2.7. Statistical Analysis

All statistical analyses were performed at raw data using GraphPad Prism (GraphPad Software Inc., San Diego, CA, USA). Where the figures show normalized data, the mean of the controls of the individual experiment was set to 1 and the error bars show the standard error calculated from the raw data. Statistical significance was determined using one- or two-way ANOVA with* post hoc* tests, or Student's *t*-test as appropriate. *P* values less than or equal to 0.05 were considered statistically significant.

## 3. Results

### 3.1. JNK Isoform Expression and Activity in INS-1 Cell

**Figure 1:**
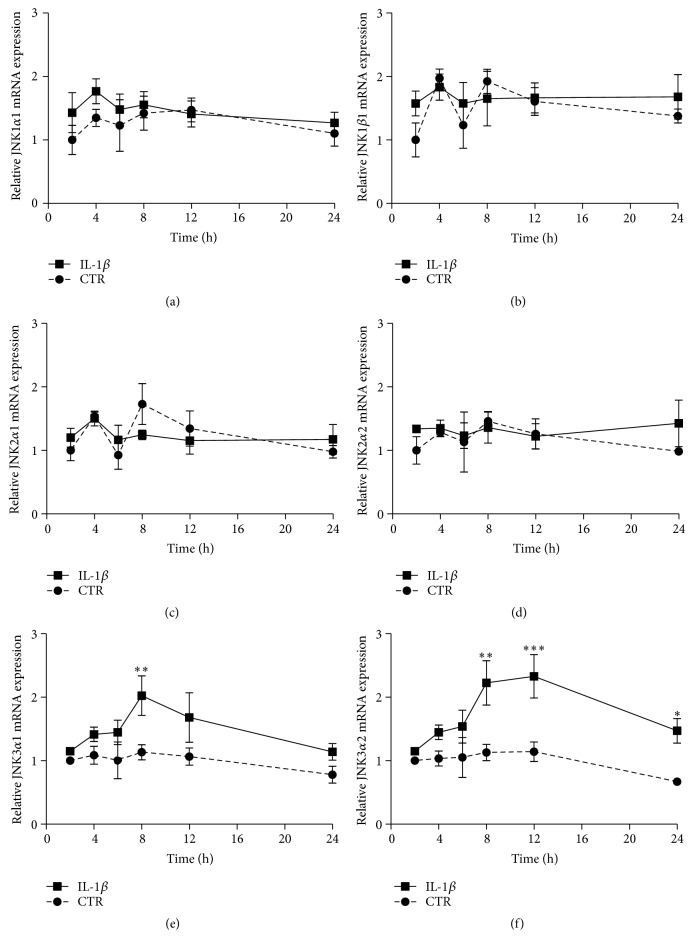
JNK isoform expression in INS-1 cell. INS-1 cells were exposed to 150 pg/mL IL-1*β* (square) or vehicle (circle) for 2 to 24 h. Relative mRNA of the JNK isoforms was measured using quantitative RT-PCR and normalized to the average of 18S and hprt1. (a) Relative JNK1*α*1 mRNA expression, (b) relative JNK1*β*1 mRNA expression, (c) relative JNK2*α*1 mRNA expression, (d) relative JNK2*β*2 mRNA expression, (e) relative JNK3*α*1 mRNA expression, and (f) relative JNK3*α*2 mRNA expression. Data are means ± SEM of *n* = 4 independent experiments. ^*∗*^ *P* < 0.05, ^*∗∗*^ *P* < 0.01, and ^*∗∗∗*^ *P* < 0.001.

The JNK family of proteins consists of more than 10 isoforms. In order to determine which isoforms are expressed in the INS-1 cell model, we created rat-specific primers and probes (Table 1), utilizing the human isoform-specific primer and probe sequences determined by Dreskin et al. [34] and modifying them based upon a cross species homology comparison between human and mouse, as the rat JNK isoform sequences have not been published. Utilizing quantitative RT-PCR, we found that the INS-1 cells expressed JNK1*α*1, JNK1*β*1, JNK2*α*1, JNK2*α*2, JNK3*α*1, and JNK3*α*2. JNK1*α*2 and JNK1*β*2 were expressed at very low levels and JNK2*β*1 and JNK2*β*2 were not expressed in INS-1 cells (data not shown). We chose to examine IL-1*β*-induced regulation of the six isoforms with the higher level of expression in the INS-1 cell line. We exposed INS-1 cells to 150 pg/mL of IL-1*β* or vehicle for 2 to 24 h. We used 150 pg/mL IL-1*β* to induce apoptosis in the INS-1 cells as titration experiments showed that this was the lowest concentration sufficient to induce maximal apoptosis (data not shown). Utilizing RT-PCR, we found that JNK1*α*1, JNK1*β*1, JNK2*α*1, and JNK2*α*2 were not regulated by IL-1*β*, whereas JNK3*α*1 was significantly upregulated at 8 h and JNK3*α*2 was upregulated from 8 to 24 h (Figures 1(a)–1(f)). Comparison of the data obtained with JNK isoform-specific primers with data obtained with nonisoform-specific Taqman primers confirmed that JNK1 and JNK2 were not regulated by IL-1*β*, whereas JNK3 was upregulated from 6 to 12 h (Supplementary Figures 1A–1C, in Supplementary Material available online at http://dx.doi.org/10.1155/2016/1312705).

**Figure 2:**
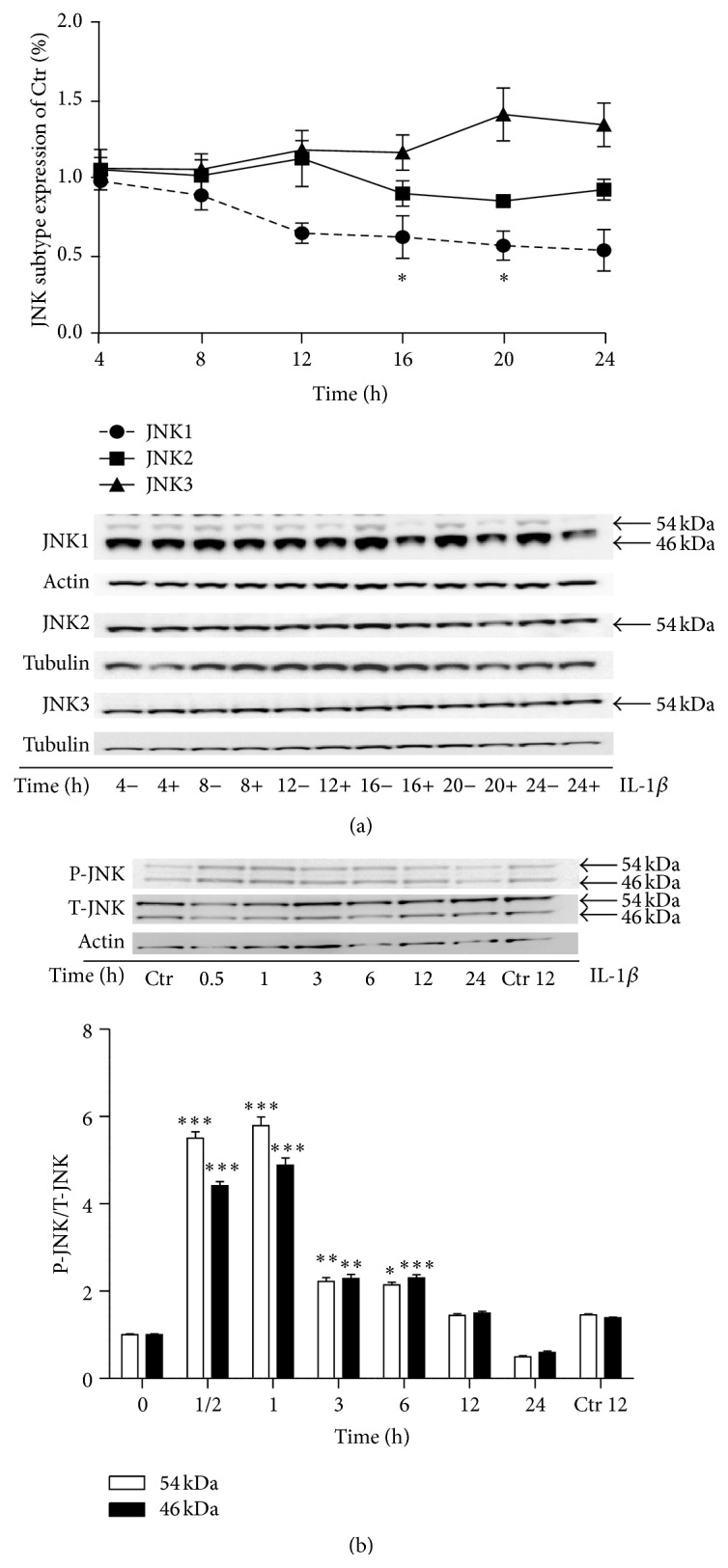
IL-1*β* reduces JNK1 but not JNK2 or JNK3 protein expression and increases P-JNK after 0.5 h of exposure in INS-1 cells. (a) INS-1 cells were exposed to 150 pg/mL IL-1*β* or vehicle for 4 to 24 h. JNK subtypes protein expression, JNK1 (circle), JNK2 (square), and JNK3 (triangle), were measured by immunoblotting and normalized to actin or tubulin. Data are shown as % IL-1*β*-regulated JNK subtype expression of their respective nonexposed controls. Data are shown as means of *n* = 3–5 independent experiments ± SEM; ^*∗*^ *P* < 0.05 versus 4 h time point. (b) INS-1 cells were exposed to 150 pg/mL IL-1*β* or vehicle for 0.5 to 24 h. INS-1 cells exposed for IL-1*β* for 12 h were lysed at an earlier time point paralleled with the 12 h vehicle ctrl. P-JNK was measured by immunoblotting and normalized to T-JNK. Data are shown as means of *n* = 4 independent experiments ± SEM; ^*∗*^ *P* < 0.05, ^*∗∗*^ *P* < 0.01, and ^*∗∗∗*^ *P* < 0.001 versus vehicle (0 h). Representative gels are shown.

We next asked if IL-1*β* altered JNK protein expression in INS-1 cells. As there are no available commercial JNK isoform-specific antibodies, we used JNK1, JNK2, or JNK3 subtype specific antibodies. We exposed INS-1 cells to 150 pg/mL IL-1*β* for 4 to 24 h to measure the expression and regulation of the JNK subtype proteins by Western blot analysis. The JNK1 subtype encompasses JNK1*α*1 and JNK1*β*1 isoforms translating into 46 kDa and JNK1*α*2 and JNK1*β*2 isoforms translating into 54 kDa isoform proteins, respectively. We found that only JNK1 46 and 54 kDa isoform proteins expressions were significantly regulated in that they were decreased by IL-1*β* in a time-dependent manner (Figure 2(a)). The JNK2 subtype includes JNK2*α*1 and JNK2*α*2 translating into 46 kDa and 54 kDa isoform proteins, respectively. The JNK2 antibody reacts only with the 54 kDa isoform protein, compatible with JNK2*α*2 being the predominating JNK2 isoform expressed in INS-1 cells (unpublished data). The JNK3 subtype specific antibody reacts only with the 54 kDa protein isoform corresponding to JNK3*α*2; however, JNK3*α*1 that translates into a 46 kDa protein isoform and JNK3*α*2 are expressed at equally high levels in INS-1 cells (unpublished data). However, the observed IL-1*β*-induced JNK3 mRNA expression did not translate into increased JNK3 54 kDa isoform protein expression (Figure 2(a)). Of note, detection of IL-1*β*-regulated JNK3*α*1 isoform protein expression was not possible with this JNK3 subtype antibody.

Finally, we exposed INS-1 cells to 150 pg/mL IL-1*β* or vehicle for 0.5 to 24 h to measure JNK phosphorylation as a measure of JNK activity. JNK 46 and 54 kDa isoforms were both significantly phosphorylated after 0.5 to 6 h of IL-1*β* exposure, with maximum peak phosphorylation of the isoforms after 0.5 to 1 h of IL-1*β* exposure (Figure 2(b)).

In summary, IL-1*β* increased JNK3*α*1 and JNK3*α*2 mRNA expression and decreased JNK1 subtype protein expression in INS-1 cells. Furthermore, IL-1*β*-induced JNK phosphorylation peaks after 0.5 to 6 h of stimulation.

### 3.2. JNK Knockdown INS-1 Cell Lines

**Figure 3:**
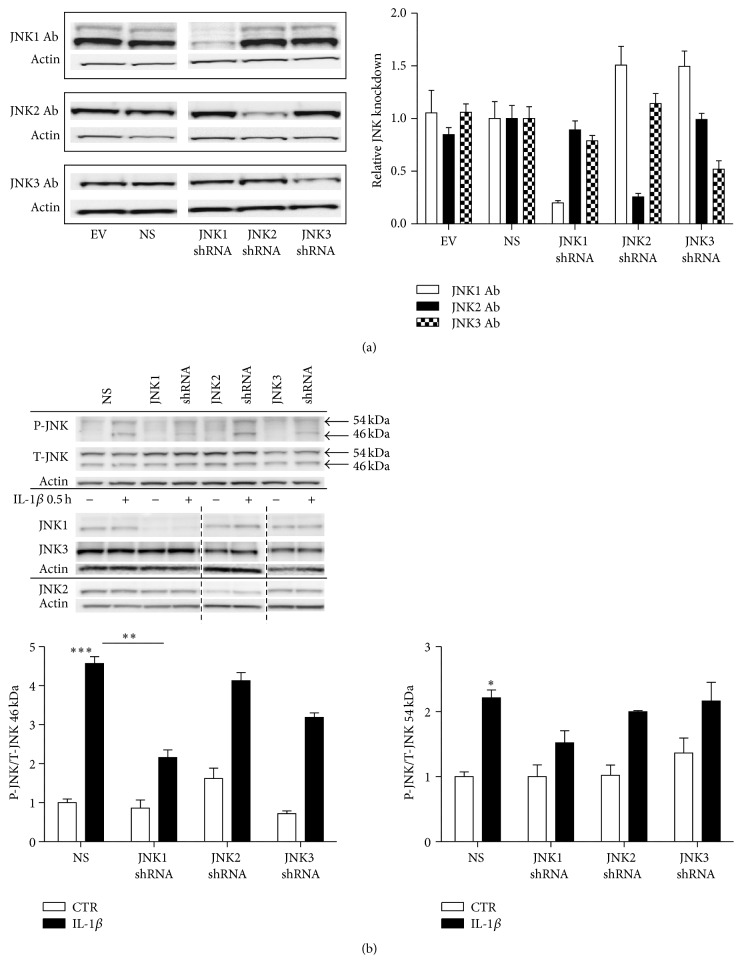
Phosphorylated JNK in individual JNK subtype knockdown INS-1 cell lines exposed to IL-1*β* for 0.5 h. INS-1 cells were transduced with Lentivirus containing shRNA directed against JNK1, JNK2, or JNK3 and nonsense (NS) or empty vector (EV) controls. Cells stably expressing the shRNA plasmids were selected with puromycin. (a) Stable INS-1 cell lines expressing shRNA for JNK1, JNK2, JNK3, nonsense shRNA, or empty vector controls were cultured for 4 days, protein was isolated, and JNK1, JNK2, and JNK3 protein knockdown expression was analyzed by immunoblotting. Data are shown as means ± SEM of *n* = 3 independent experiments. Representative blots are shown. (b) Stable INS-1 cell lines expressing shRNA for JNK1, JNK2, JNK3, nonsense shRNA, or empty vector were exposed to 150 pg/mL IL-1*β* (closed bars) or vehicle (open bars) for 0.5 h. P-JNK was assessed with immunoblotting and normalized to T-JNK. Data were quantified in respect to 46 kDa and 54 kDa P-JNK/T-JNK. Data are shown as means ± SEM of *n* = 3 independent experiments. Specific knockdowns of the individual JNK subtypes, JNK1, JNK2, or JNK3, are shown with their respective actin. Blots were cut as indicated by the dotted black line. ^*∗*^ *P* < 0.05, ^*∗∗*^ *P* < 0.01, and ^*∗∗∗*^ *P* < 0.001 versus IL-1*β* exposed NS. Representative gels are shown.

To investigate the differential roles of the JNK subtypes in IL-1*β*-induced cell death, we produced stable INS-1 knockdown cell lines expressing shRNAs directed against JNK1, JNK2, or JNK3, as well as nontarget (nonsense, NS) shRNA and empty vector (EV) controls. The JNK knockdown cell lines were tested for knockdown specificity and efficiency by Western blot analysis using JNK subtype specific antibodies (Figure 3(a)). We achieved specific knockdown of the intended JNK subtypes by approximately ~80% JNK1, ~75% JNK2, and ~45% JNK3 knockdown compared to control NS (NS shRNA) INS-1 cells (Figure 3(a)).

We next wished to measure early response JNK activity in the stable JNK knockdown INS-1 cell lines to investigate how knockdown of the individual JNK subtype affected total phosphorylation status of the different JNK isoforms (46 or 54 kDa). We exposed the stable JNK knockdown INS-1 cells to 150 pg/mL IL-1*β* or vehicle for 0.5 h (Figure 3(b)) as we detected peak JNK phosphorylation at this time point in nontransduced INS-1 cells (Figure 2(b)). Though the JNK knockdown INS-1 cell lines showed specific JNK1, JNK2, or JNK3 knockdown (Figure 3(b)), this did not affect the total level of JNK (all JNK subtypes) (Figure 3(b)). Interestingly, JNK1 knockdown (JNK1 shRNA) INS-1 cells showed significantly reduced IL-1*β*-induced phosphorylation of the 46 kDa isoforms compared to control NS expressing INS-1 cells. Phosphorylations of the 46 kDa isoforms in JNK2 knockdown (JNK2 shRNA) and JNK3 knockdown (JNK3 shRNA) INS-1 cells were not different compared to the control NS INS-1 cells (Figure 3(b)). There were no differences in phosphorylation of the 54 kda JNK isoforms between 0.5 h IL-1*β* exposed NS and JNK1, JNK2, or JNK3 knockdown INS-1 cell lines (Figure 3(b)). To summarize, only JNK1 knockdown reduced early IL-1*β*-mediated activity of the 46 kDa JNK isoforms as determined by phosphorylation in INS-1 cells.

### 3.3. JNK1 Knockdown Attenuates IL-1*β*-Induced Caspase 3 Activation and Apoptosis in INS-1 Cells

**Figure 4:**
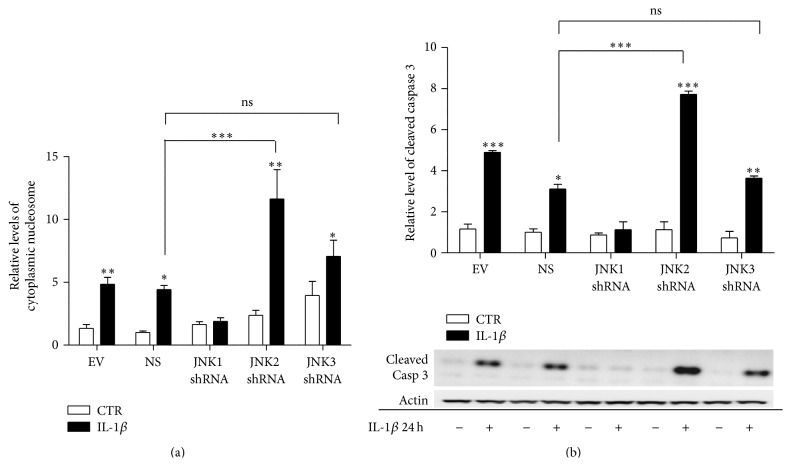
JNK1 knockdown attenuates IL-1*β*-induced caspase 3 activation and apoptosis in INS-1 cells. Stable INS-1 cell lines expressing shRNA for JNK1, JNK2, JNK3, nonsense shRNA, or empty vector controls were exposed to 150 pg/mL IL-1*β* (closed bars) or vehicle (open bars) for 24 h. (a) Apoptosis was measured as the relative levels of cytoplasmic nucleosomes in INS-1 stable cell lines lysates using the Roche Cell Death detection Elisa kit. Data are shown as means ± SEM of *n* = 5 independent experiments. (b) Cleaved caspase 3 was assessed with immunoblotting and normalized to actin. Data are shown as means ± SEM of *n* = 4 independent experiments; ^*∗*^ *P* < 0.05, ^*∗∗*^ *P* < 0.01, and ^*∗∗∗*^ *P* < 0.001, comparing caspase 3 activity in the presence with the activity in the absence of IL-1*β*, unless otherwise indicated. Representative gels are shown.

Next, to investigate the functional impact of knockdown of the individual JNK subtypes, we exposed the stable JNK knockdown and control cell lines to 150 pg/mL of IL-1*β* or vehicle for 24 h, after which apoptosis markers were measured by Elisa and Western blotting. IL-1*β*-induced apoptosis was observed in the control NS and EV expressing INS-1 cells. In contrast, knockdown of JNK1 prevented IL-1*β*-induced apoptosis, measured as decreased levels of cytoplasmic nucleosomes (Figure 4(a)). JNK1 knockdown also attenuated IL-1*β*-induced caspase 3 cleavage (Figure 4(b)). Interestingly, knockdown of JNK2 significantly potentiated IL-1*β*-induced apoptosis and caspase 3 activation, while JNK3 knockdown did not significantly affect IL-1*β*-induced apoptosis or cleavage of caspase 3 compared to NS cells.

Since the JNK members had differential effects on IL-1*β*-induced apoptosis, we next determined if there were unique early response gene clusters regulated by each JNK member. INS-1 cells stably expressing shRNAs directed against JNK1, JNK2, JNK3, or NS were exposed to 150 pg/mL of IL-1*β* or vehicle for 45 min. mRNA expression was analyzed by microarray analysis. When we compared the normalized gene expression profile of the 45 min IL-1*β* exposed JNK1, JNK2, or JNK3 knockdown INS-1 cells with the gene expression profile of 45 min IL-1*β* exposed NS INS-1 cells, adjusted for their IL-1*β* nonexposed control conditions, 74, 144, and 134 genes, respectively, were found to be differentially regulated (Supplementary Tables 1–3). The JNK1 knockdown INS-1 cell line gene expression pattern was significantly different from that of JNK2 or JNK3 knockdown and NS INS-1 cells (Supplementary Figure 2). However, overall, there were no significant clusters or pathway associations to cast light on the potential pathways or early IL-1*β*-induced mechanisms underlying the unique actions of the JNK subtypes, and we therefore decided to proceed with a candidate gene approach to understand the mechanisms of action of the JNK subtypes.

### 3.4. JNK1 Knockdown Attenuates the Increase of Myc mRNA Expression by IL-1*β*

**Figure 5:**
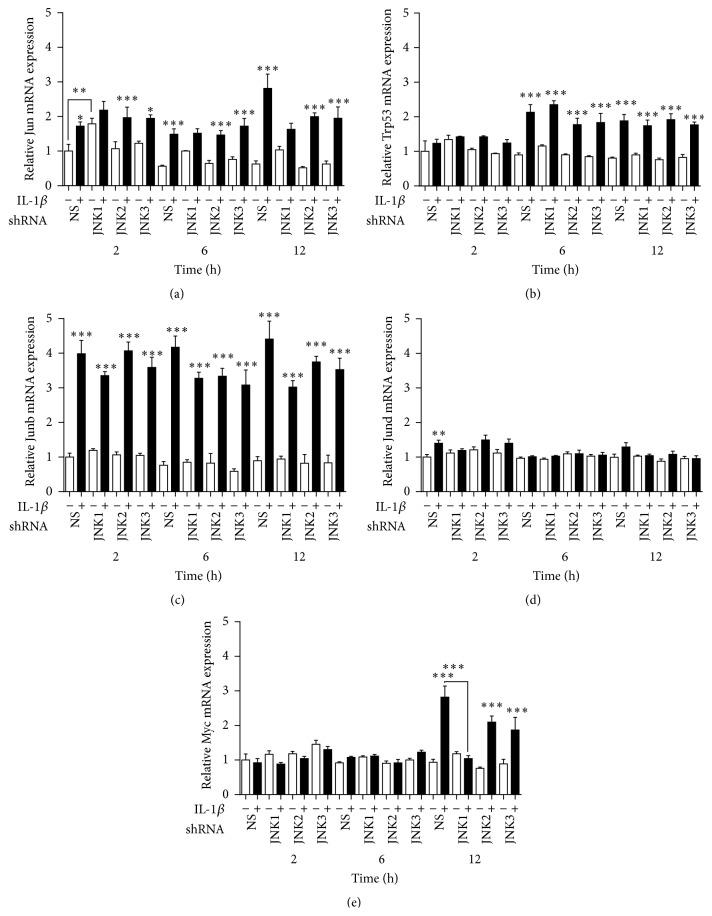
JNK1 knockdown attenuates the regulation of Myc mRNA expression by IL-1*β*. Stable INS-1 cell lines expressing shRNA for JNK1, JNK2, JNK3, nonsense shRNA, or empty vector controls were exposed to 150 pg/mL IL-1*β* (closed bars) or vehicle (open bars) for 2, 6, and 12 h. Relative mRNA expression were measured using quantitative RT-PCR and normalized to the average of 18S and Hprt1. (a) Relative Jun mRNA expression, (b) relative Trp53 mRNA expression, (c) relative Junb mRNA expression, (d) relative Jund mRNA expression, and (e) relative Myc mRNA expression. Data are shown as means ± SEM of 3–5 independent experiments. ^*∗*^ *P* < 0.05, ^*∗∗*^ *P* < 0.01, and ^*∗∗∗*^ *P* < 0.001, comparing expression in the presence with expression in the absence of IL-1*β*, unless otherwise indicated.

We therefore next analyzed in the JNK knockdown cells specific JNK-related signalling molecules known to be regulated by the JNK proteins to determine if the regulation of these genes by IL-1*β* is mediated specifically by JNK1. INS-1 cells stably expressing shRNA directed against JNK1, JNK2, or JNK3, or the NS or EV controls, were exposed to 150 pg/mL of IL-1*β* for 2, 6, and 12 h. IL-1*β* upregulated Jun mRNA from 2 to 12 h, Trp53 mRNA at 6 and 12 h, Junb mRNA from 2 to 12 h, Jund mRNA at 2 h, and Myc mRNA at 12 h (Figures 5(a)–5(e)). These findings indicate that many of the common JNK-related signalling proteins are regulated by IL-1*β*. Interestingly, there was a temporal difference in the regulation of the mRNA transcripts, as Jun and Junb were upregulated at all time points, whereas Myc was upregulated only at 12 h and Jund was upregulated significantly only at 2 h.

Knockdown of JNK1, JNK2, or JNK3 did not affect the regulation of Trp53 or Junb mRNA expression by IL-1*β*. This indicates that these mRNA transcripts are not regulated by JNK1, JNK2, or JNK3 specifically, either due to redundancy in the actions of the JNK members or due to the fact that they are not regulated by the JNK proteins in INS-1 cells. JNK1 knockdown increased basal Jun mRNA expression at 2 h (Figure 5(a)) and attenuated the upregulation of Myc by IL-1*β* at 12 h (Figure 5(e)), indicating that JNK1 may be involved in the basal regulation of Jun and that it specifically mediates the regulation of Myc mRNA expression by IL-1*β*.

### 3.5. JNK1 Knockdown Attenuated the IL-1*β*-Induced Increase of Myc Protein Levels

**Figure 6:**
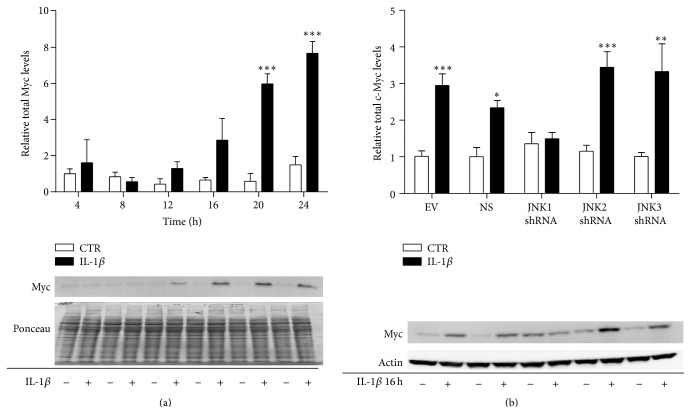
JNK1 knockdown prevents the regulation of total Myc protein by IL-1*β*. INS-1 cells were exposed to 150 pg/mL IL-1*β* (closed bars) or vehicle (open bars) for 4 to 24 h. Protein was isolated and (a) total Myc protein levels were analyzed by immunoblotting. Total protein was used as the loading control. Data are shown ± SEM of *n* = 3-4 independent experiments. INS-1 cells stably expressing shRNA directed against JNK1, JNK2, JNK3, or the nonsense or empty vector controls were exposed to 150 pg/mL IL-1*β* (black bars) or left unexposed (white bars) for 16 h. Protein was isolated, and (b) total Myc protein levels were analyzed by immunoblotting. Actin was used as the loading control. Data are shown ± SEM of *n* = 5 independent experiments. ^*∗∗*^ *P* < 0.01 and ^*∗∗∗*^ *P* < 0.001 comparing Myc levels in the presence with Myc levels in the absence of IL-1*β*, unless otherwise indicated.

Next, we wished to determine if IL-1*β* regulates the levels of Myc protein and if JNK1 mediates this regulation in response to IL-1*β*. INS-1 cells were exposed to 150 pg/mL of IL-1*β* or vehicle for 4 to 24 h, and protein was analyzed by Western blot analysis. IL-1*β* increased the total amount of Myc protein from 12 to 24 h (Figure 6(a)). The increase in total Myc protein by IL-1*β* corresponded to the timing of the upregulation of Myc mRNA by IL-1*β*, which we noted at 12 h (Figure 5(e)). Next, we exposed the INS-1 cells stably expressing shRNA directed against JNK1, JNK2, or JNK3, or the NS or EV controls to 150 pg/mL of IL-1*β* or vehicle for 16 h. Knockdown of JNK1 prevented IL-1*β* induction of total Myc protein expression (Figure 6(b)). This indicates that JNK1 mediates IL-1*β* regulation of total Myc protein levels. These results, taken together with the data in Figure 5(e), indicate that JNK1 mediates inflammatory regulation of Myc transcription and translation in INS-1 cells.

### 3.6. Myc Knockdown Attenuates IL-1*β*-Induced Apoptosis in INS-1 Cells

**Figure 7:**
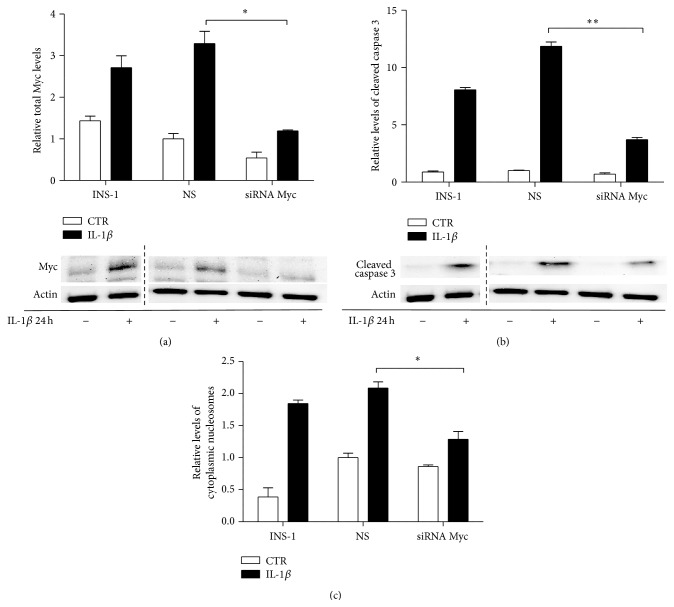
Myc knockdown attenuates IL-1*β*-induced apoptosis in INS-1 cells. INS-1 cells were transfected with siRNA directed against Myc, a scrambled siRNA control (NS) or vehicle (INS-1) for 16 h. Thereafter, cells were exposed to 150 pg/mL IL-1*β* (closed bars) or vehicle (open bars) for 24 h. (a) Total Myc protein levels and (b) cleaved caspase 3 were analyzed by immunoblotting. Actin was used as the loading control. Data are shown as means ± SEM of 3-4 independent experiments. Blots shown are representative. (c) Apoptosis was measured as relative levels of cytoplasmic nucleosomes in lysates measured by the Roche Cell Death Elisa kit. Data are shown as means ± SEM of 3-4 independent experiments. ^*∗*^ *P* < 0.05 and ^*∗∗*^ *P* < 0.01 versus IL-1*β* exposed NS.

The Myc protein has been shown previously to be involved in JNK-mediated apoptosis triggered by UV irradiation or antineoplastic drugs [26, 35]. We therefore wished to determine if Myc mediates IL-1*β*-induced apoptosis in the INS-1 *β*-cell model. INS-1 cells were transiently transfected with either siRNA directed against Myc or a control NS oligonucleotide sequence and then exposed to 150 pg/mL of IL-1*β* or vehicle for 24 h (Figure 7). Transfection with siRNA directed against Myc decreased the total level of Myc by ~2.8-fold detected by Western blot analysis (Figure 7(a)) and, interestingly, attenuated the IL-1*β*-induced caspase 3 activation by ~3.2-fold (Figure 7(b)). In addition, transfection of INS-1 cells with Myc siRNA attenuated IL-1*β*-induced apoptosis by ~1.6-fold (Figure 7(c)). This indicates that Myc may mediate IL-1*β*-induced apoptosis in the INS-1*β*-cell line and, taken together with the previous results, that the JNK1-Myc pathway is an important mediator of IL-1*β*-induced apoptosis.

## 4. Discussion

Few studies have investigated the differential roles of the JNK subtypes in cellular apoptosis, or if there are specific downstream signaling proteins utilized by a specific JNK subtype to mediate its actions. It is known that IL-1*β* does not induce apoptosis in most cells but the pancreatic *β*-cells belong to the exceptions. Many studies have shown that JNK plays an important role in cellular apoptosis and specifically in cytokine and IL-1*β*-induced *β*-cell apoptosis [19] most using the nonspecific JNK inhibitor SP600125 [36]. We show here that JNK1*α*1, JNK1*β*1, JNK2*α*1, JNK2*α*2, JNK3*α*1, and JNK3*α*2 isoforms are expressed in INS-1 cells whereas only the JNK3 isoforms mRNA is regulated by IL-1*β*. When we analyzed JNK1, JNK2, and JNK3 subtype protein expression, only JNK1 subtype was significantly downregulated by IL-1*β* after 24 h of exposure. We believe that this downregulation of JNK1 subtype is not mediated by proteases after cell lysis since we use a broad protease inhibitor cocktail in our lysis buffer. The reduced JNK1 subtype expression may though be a side effect of apoptosis due to caspase 3-mediated cleavage; however, in other stress-induced apoptotic cell lines, JNK protein expression is shown to be caspase-independent [37, 38]. Gene expression profiling studies followed by functional characterization of candidate genes have demonstrated that the exposure of insulin-producing cell lines, fluorescence-activated cell-sorted primary rodent *β*-cells, intact rodent islets, and human islets to proinflammatory cytokines induces not only upregulation of proapoptotic/downregulation of antiapoptotic genes, but also a protective response including downregulation of proapoptotic/upregulation of antiapoptotic genes, with a high degree of concordance between the different model systems [39, 40]. These findings suggest that inflammatory attack on the pancreatic *β*-cell induces a race between deleterious and protective responses, of which the deleterious pathways eventually prevail [41]. We believe that the observed decrease in JNK1 content following 16 h of exposure to IL-1*β* is a late compensatory negative feedback mechanism serving to suppress signaling via JNK1; however, this late suppression in JNK1 content is insufficient to prevent apoptosis.

We selectively knocked down the three JNK subtypes to understand their individual contributions. Knockdown of JNK1 completely prevented IL-1*β*-induced INS-1 cell apoptosis, while JNK2 knockdown potentiated cytokine-induced INS-1 cell apoptosis. This is an interesting observation as JNK2 has been shown to inhibit JNK1 signaling [42], and thus knockdown of JNK2 may act to increase JNK1 activity. Knockdown of JNK3 did not significantly affect IL-1*β*-induced apoptosis compared to NS cells but an increase could be observed, but not in levels of cleaved caspase 3. JNK3 has been suggested as being protective against cytokine-induced INS-1E cell death [15]. Both JNK1 and JNK2 knockdown protected INS-1E cells against apoptosis by 40 and 60%, respectively, in that study. We found complete protection of apoptosis by JNK1 knockdown and found that JNK2 knockdown potentiated the effect of IL-1*β*. Our study and [15] differed in the following respects: we utilized INS-1 cells stably expressing shRNA, whereas Abdelli et al. used transiently siRNA transfected INS-1E cells. However, the key difference between these studies is the inflammatory stimulus used. Abdelli et al. used a cytokine combination, containing 10 ng/mL IL-1*β*, 25 ng/mL TNF-*α*, and 150 ng/mL IFN-*γ*, while we exposed INS-1 cells to only IL-1*β* at 150 pg/mL. TNF-*α* receptor is a death-domain containing receptor and activates caspase 8 directly. The TNFR also signals through JNK and NF*κ*B, as does the IL-1 receptor. IFN-*γ* signals through the JAK-STAT pathway. Thus, using a cytokine combination, confounding pathways and downstream signaling molecules are activated compared to testing IL-1*β* alone. However, as we only achieved ~50% knockdown of JNK3 in this study, new studies with more efficient JNK3 knockdown might acknowledge JNK3 as a protective protein.

We also investigated early IL-1*β*-induced JNK phosphorylation as a surrogate for JNK activity in the JNK knockdown INS-1 cells. Here, we only observed significant reduction of phosphorylation of the 46 kDa isoform in 0.5 h IL-1*β* exposed JNK1 knockdown INS-1 cells when compared to NS INS-1 cells. This was not surprising to us as the major JNK1 isoforms expressed in INS-1 cells translate into 46 kDa proteins and as we achieved a high JNK1 knockdown efficiency in the JNK1 knockdown INS-1 cell line. Despite high specific knockdown of the individual JNK subtypes in the shRNA expressing INS-1 cell lines, we did not see any changes in total levels of JNK. The total JNK antibody recognizes all three JNK subtypes and therefore a reduction in only one subtype is unlikely to affect the total amount of JNK. The reduced 46 kDa phosphorylation might explain the decreased IL-1*β*-induced apoptosis in JNK1 knockdown INS-1 cells as inhibition of total JNK activity decreases intracellular JNK signalling and improves rodent *β*-cell survival* in vitro* in response to cytokines [5, 18, 19, 43]. We did not observe altered 54 kDa isoform phosphorylation between 0.5 h IL-1*β* exposed NS and JNK1, JNK2, or JNK3 perhaps indicating that IL-1*β*-induced phosphorylation in the individual JNK knockdown subtype INS-1 cells is covered by the remaining JNK isoforms.

There are many potential downstream targets of JNK1 that activate apoptosis, such as the mitochondrial stress pathway and caspase activation, but there are few studies analyzing if there are specific signaling proteins or transcription factors that are uniquely regulated by each JNK subtype. We performed microarray mRNA expression profiling to investigate the genes regulated by the JNK subtypes. There were no differences in basal gene expression levels in JNK1, JNK2, or JNK3 shRNA expressing INS-1 cells when compared to NS shRNA expressing INS-1 cells, indicating that individual JNK subtype knockdown does not initiate differential basal gene regulation. Exposure of the cells to IL-1*β* for 45 min initiated differential gene regulation; however, no significant clusters were identified. This indicates that the JNK subtypes might mediate their major differential effect by modulating activity of proteins rather than affecting early IL-1*β*-induced gene transcription in *β*-cells. Using a transcription factor candidate gene expression analysis, we found that Myc was specifically regulated by JNK1 after 12 h of IL-1*β* exposure. We found that Myc mRNA and total protein levels were specifically downregulated in JNK1 knockdown INS-1 cells and that knockdown of Myc decreased IL-1*β*-induced apoptosis and cleaved caspase 3. In addition to activating Myc expression, JNK proteins are known to increase the stability of the Myc protein [44] that normally has a short half-life of approximately 30 minutes. Our data also suggests that JNK1 increases the stability of total Myc, as Myc protein levels were increased in wild-type INS-1 cells by IL-1*β* from 16 h to 24 h; additionally, we found that Myc mRNA expression was increased at 12 h. This increase could be due to an increased stability of the mRNA transcript, as opposed to transcriptional activation, given that there is a delayed increase in Myc mRNA expression relative to other JNK-regulated genes, such as Jun, Junb, and Jund, which were all increased at 2 h.

Myc is involved in mediating numerous cellular functions, including cell proliferation, growth, differentiation, and apoptosis [45]. There is much debate as to how Myc regulates these opposing cellular processes, but it seems to be through a complex and intricate balance between Myc and its protein interaction partner MAX, as well as other signaling pathways [45]. There are also numerous pathways through which Myc can promote cellular death. Myc induces the expression of the tumor suppressor protein cyclin-dependent kinase inhibitor 2A, which stabilizes p53, an important regulator of apoptosis, by sequestering the E3 ubiquitin-protein ligase, mouse double minute 2 homolog, in turn degrading p53 [46]. Myc also induces the proapoptotic BH3-only protein, Bim, and blocks the expression of the antiapoptotic proteins Bcl-2 and Bcl-xl. Myc has been shown to promote intrinsic cell death by destabilizing via the Bax protein [47]. Thus, Myc knockdown in cisplatin-induced apoptosis in A549 human lung carcinoma cells blocked cytochrome C release and prevented Bax oligomerization [48]. Another potential death promoting mechanism is through the p53-p21 pathway. Myc binds to the cell cycle gene p21CIP1 promoter, thereby preventing p53-induced transcription [49]. p53 induction of p21CIP1 drives the cell into senescence, and blocking p21 upregulation redirects the actions of p53 to activate apoptotic pathways [49]. p53 was significantly upregulated in INS-1 cells following IL-1*β* treatment; however, when we analyzed p21CIP1 mRNA expression in the INS-1 cells stably expressing JNK1 shRNA, we did not observe a decrease in expression, and thus we do not favor this mechanism of action.

In nonstressed islets, Myc expression is low [50] and *β*-cell specific Myc overexpression markedly increases *β*-cell apoptosis [51]. Here, we suggest that Myc is a key mediator of IL-1*β*-induced apoptosis in the INS-1 cell model. We additionally propose that JNK1 specifically regulates Myc, implicating Myc as a potential downstream relay of the apoptotic effect of JNK1. Further experiments are likewise required to verify the direct involvement of Myc in mediating the apoptotic actions of JNK1, such as overexpression of JNK1 combined with Myc knockdown.

## 5. Conclusion

In summary, this study suggests that JNK1 is a key mediator of IL-1*β*-induced INS-1 cell apoptosis, that the transcription factor Myc is regulated specifically by JNK1, and that Myc may mediate the apoptotic actions of JNK1. Future studies are required to investigate if the JNK1-Myc pathway is involved in cytokine-induced apoptosis in other cell types to determine if this is a pancreatic *β*-cell specific effect or a more general cellular effect. We propose that the JNK1-Myc pathway may be a target for protecting pancreatic *β*-cells from inflammatory stress, and thus a potential treatment target in diabetes.

## Supplementary Material

Figure S1: JNK subtype mRNA expression and regulation by IL-1*β* in INS-1 cell.
Figure S2: JNK subtypes differentially regulate INS-1 cell gene expression.
Table S1: List of normalized gene expression profile of the 45 min IL-1*β* exposed JNK1 knockdown (KD) INS-1 cells.
Table S2: List of normalized gene expression profile of the 45 min IL-1*β* exposed JNK2 knockdown (KD) INS-1 cells.
Table S3: List of normalized gene expression profile of the 45 min IL-1*β* exposed JNK3 knockdown (KD) INS-1 cells.

## Figures and Tables

**Table 1:** Rat JNK isoform-specific primers.

Isoform	Sense primer (5′-3′)	Antisense primer (5′-3′)	Probe
JNK1*α*1	GAGAAATGGTTTGCCACA	ACTGCTGCACCTGTGCTA	(VIC)-TTGAACAGCTCGGAACACCTTGTCCTG-(TAMRA)

JNK1*α*2	GAGAAATGGTTTGCCACA	ACTGCTGCACCTAAAGGA	(VIC)-TTGAACAGCTCGGAACACCTTGTCCTG-(TAMRA)

JNK1*β*1	GGAGAAATGATCAAAGGTG	ACTGCTGCACCTGTGCTA	(VIC)-TTGAACAGCTCGGAACACCTTGTCCTG-(TAMRA)

JNK1*β*2	GGAGAAATGATCAAAGGTG	ACTGCTGCACCTAAAGGA	(VIC)-TTGAACAGCTCGGAACACCTTGTCCTG-(TAMRA)

JNK2*α*1	GAGAGCTGGTGAAAGGTT	TTACTGCTGCATCTGTGC	(VIC)-AAAGTTATTGAACAGCTAGGAACACCATCC-(TAMRA)

JNK2*α*2	GAGAGCTGGTGAAAGGTT	ACTGCTGCATCTGAAGGC	(VIC)-AAAGTTATTGAACAGCTAGGAACACCATCC-(TAMRA)

JNK2*β*1	GAAATGGTCCTCCATAAAG	TTACTGCTGCATCTGTGC	(VIC)-AAAGTTATTGAACAGCTAGGAACACCATCC-(TAMRA)

JNK2*β*2	GAAATGGTCCTCCATAAAG	ACTGCTGCATCTGAAGGC	(VIC)-AAAGTTATTGAACAGCTAGGAACACCATCC-(TAMRA)

JNK3*α*1	GCCCTCACCTTCAGCACAG	AGGCAGGCGGCTAGTCAC	(VIC)-AGCAGTGAGAGTCTCCCTCCATCCTCGT-(TAMRA)

JNK3*α*2	GCCCTCACCTTCAGGTG	AGGCAGGCGGCTAGTCAC	(VIC)-AGCAGTGAGAGTCTCCCTCCATCCTCGT-(TAMRA)
