# Supplementary material for: JNK1 Deficient Insulin-Producing Cells Are Protected against Interleukin-1β-Induced Apoptosis Associated with Abrogated Myc Expression
Source: J Diabetes Res. 2016 Jan 10;2016:1312705. doi: 10.1155/2016/1312705 (PMC4745310; doi:10.1155/2016/1312705)
Supplement: Supplementary file 1 — Figure S1: JNK subtype mRNA expression and regulation by IL-1β in INS-1 cell. Figure S2: JNK subtypes differentially regulate INS-1 cell gene expression. Table S1: List of normalized gene expression profile of the 45 min IL-1β exposed JNK1 knockdown (KD) INS-1 cells. Table S2: List of normalized gene expression profile of the 45 min IL-1β exposed JNK2 knockdown (KD) INS-1 cells. Table S3: List of normalized gene expression profile of the 45 min IL-1β exposed JNK3 knockdown (KD) INS-1 cells. [file 1312705.f1.pdf]

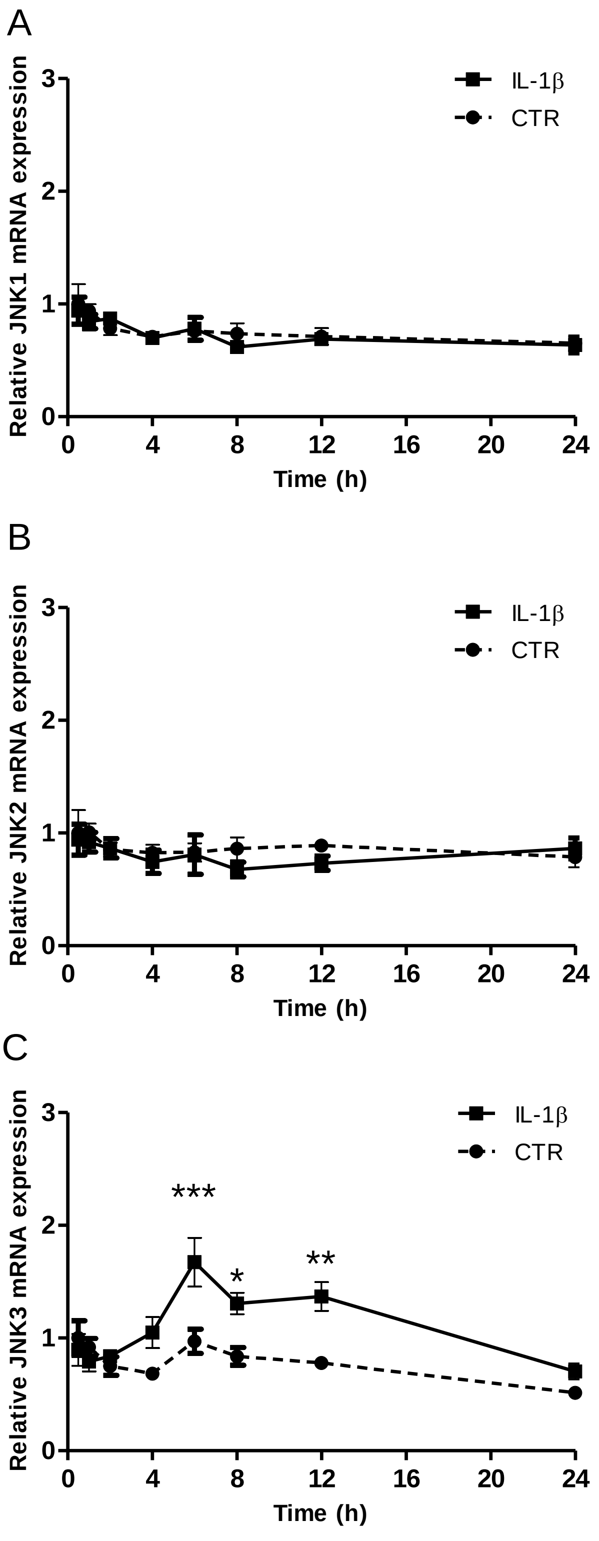

Supplementary figure 1. JNK subtype expression and regulation by IL-1β in INS-1 cell. INS-1 cells were exposed to 150 pg/ml IL-1β (square) or vehicle (circle) for 2 to 24 h. Relative mRNA of the JNK subtypes were measured using quantitative RT-PCR and normalized to the average of 18S and hprt1. A: Relative JNK1 subtype mRNA expression, B: Relative JNK2 subtype mRNA expression, C: Relative JNK3 subtype. Data are shown with ± SEM of n=4 independent experiments. *P<0.05, ** P<0.01, *** P<0.001.

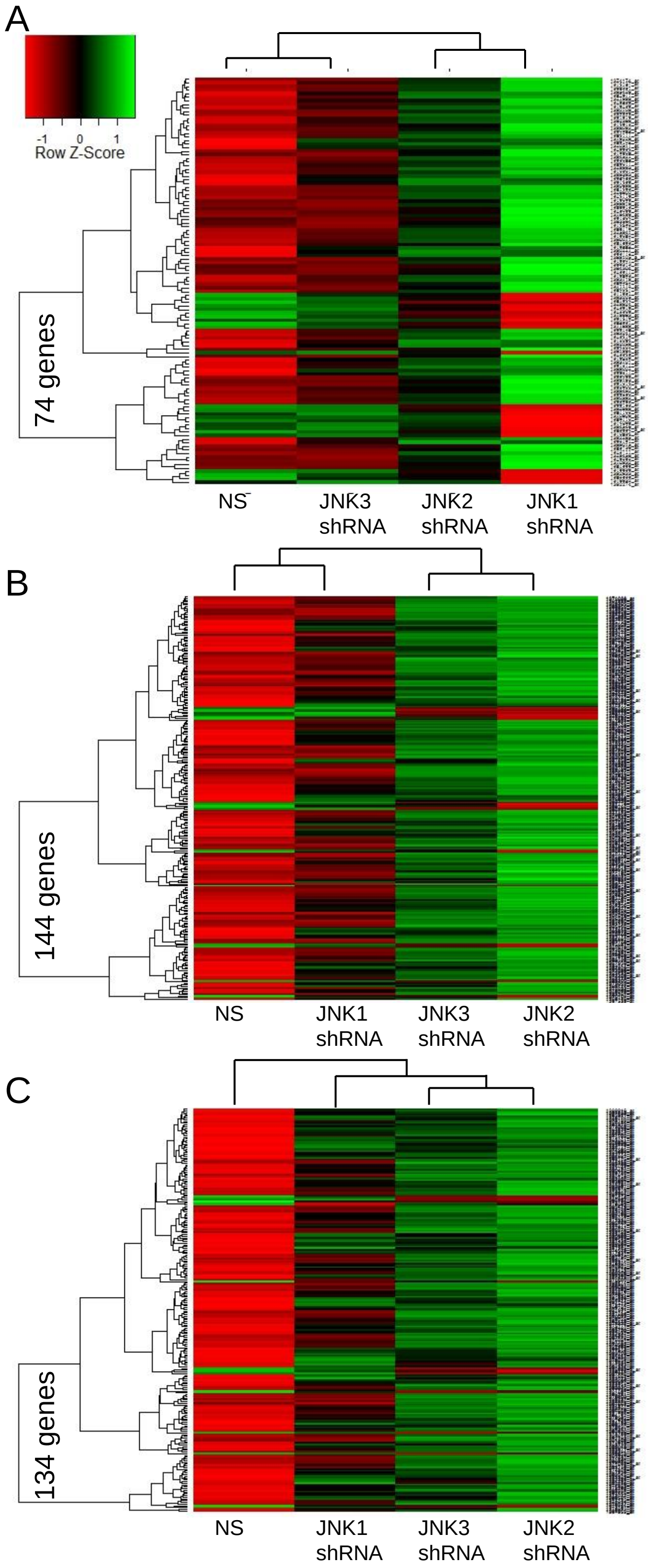

Supplementary figure 2. JNK subtypes differentially regulate INS-1 cell gene expression. Stable INS-1 cell lines expressing shRNA for JNK1, JNK2, JNK3 or non-sense shRNA were exposed to 150 pg/ml IL-1β for 45 min. mRNA expression was analyzed by microarray analysis. The heat-plot panels show clusters based on the mean log2 expression values on the y-axis for each significant probe of three independent experiments. The significantly regulated probes were selected by the following criteria: log2 fold change > 1 or log2 fold change < -1 and P-value < 0.05. A: JNK1 vs. NS 0 - 45 min. B: JNK2 vs. NS 0 - 45 min. C: JNK3 vs. NS 0 - 45 min.

ID	logFC	AveExpr	t	P.Value	adj.P.Val	B	Diff JNK1 genes	Fold Change
1370932_at	2,807600249	4,543519299	2,066925593	0,048227764	0,871062203	-4,321179758	Lrp4	7,001190433
1397304_at	2,588518358	7,545268918	2,301586117	0,029117685	0,871062203	-4,242587108	Igtp	6,014806634
1371951_at	2,224031123	8,927565744	2,517698938	0,017897953	0,871062203	-4,167191584	Fhl2	4,671970365
1369313_at	2,209742168	8,140463561	2,500803041	0,018605166	0,871062203	-4,173170901	Fhl2	4,625925937
1378739_at	2,075039328	7,156827251	2,472032701	0,019868998	0,871062203	-4,183322254	Cidec	4,213559003
1375066_at	2,030431841	7,935829401	2,145521969	0,04084802	0,871062203	-4,2952977	RGD1563319	4,085271162
1381014_at	1,962081771	5,821921042	2,183157968	0,037685271	0,871062203	-4,282739425	Ifi44	3,896237904
1383564_at	1,949158848	7,676327564	2,057576079	0,049180009	0,871062203	-4,324226202	Irf7	3,861493246
1376501_at	1,903132595	8,350255033	2,209716912	0,035587473	0,871062203	-4,273816535	Arhgap8	3,740244534
1396163_at	1,860009487	5,812309399	2,087987053	0,046142291	0,871062203	-4,314291355	Igtp	3,630100492
1391463_at	1,77393993	7,518107464	2,149225219	0,040526612	0,871062203	-4,294066594	Ddx58	3,419866315
1374718_at	1,750045726	7,431761314	2,378970257	0,024517421	0,871062203	-4,215877505	Dtx3l	3,363692271
1385426_at	1,74116007	7,09628337	2,5472419	0,016720751	0,871062203	-4,156706307	Ccdc109b	3,343038731
1374452_at	1,721788378	7,297897568	2,104276974	0,044584619	0,871062203	-4,308939485	Pde9a	3,298450326
1369153_at	1,582719405	5,587450632	2,16901538	0,038847046	0,871062203	-4,287470515	Nphs1	2,995339239
1379663_at	1,545606423	7,795924846	2,296627178	0,029437657	0,871062203	-4,244286593	Cachd1	2,919267517
1398217_at	1,502619554	6,065479288	3,352827512	0,002332805	0,871062203	-3,862549056	Zbtb41	2,833567468
1390698_at	1,446867654	7,585271522	2,350695319	0,026115309	0,871062203	-4,225676994	Mapk1ip1l	2,726155119
1376066_at	1,444511508	7,433290131	2,162103489	0,039426431	0,871062203	-4,289777499	Rnd3	2,721706516
1377698_at	1,432881187	6,767088634	2,190628019	0,037084293	0,871062203	-4,28023474	Cd40	2,699853614
1368813_at	1,411724171	7,532551488	2,088924843	0,046051324	0,871062203	-4,313983819	Cebpd	2,660549363
1375941_at	1,398490276	8,461744869	2,079816552	0,046941625	0,871062203	-4,316967815	Baiap2l1	2,636255639
1372111_at	1,374944911	4,474841359	2,622047571	0,01405314	0,871062203	-4,129996107	Cav1	2,593580072
1379458_at	1,333805222	4,247982918	2,136455196	0,041644569	0,871062203	-4,298307551	Klhl14	2,520666445
1369105_a_at	1,312150221	8,459135664	2,180253369	0,037921301	0,871062203	-4,283712261	Pkib	2,483113525
1383247_a_at	1,301953506	9,698403711	2,454513886	0,02067681	0,871062203	-4,189484299	Spns2	2,46562519
1396250_at	1,295919845	5,703022287	2,846911904	0,008231589	0,871062203	-4,04858723	Coro1c	2,455334947
1382791_at	1,276702579	4,700106143	2,318941253	0,028022561	0,871062203	-4,236627497	Zfp617	2,422845784
1372064_at	1,263820434	9,429614879	2,222608745	0,034607563	0,871062203	-4,269467734	Cxcl16	2,401307956
1398393_at	1,25540626	7,594048504	2,497392106	0,01875103	0,871062203	-4,174376431	Mapk11	2,387343663
1370307_at	1,251121258	7,549983008	2,134740442	0,041796766	0,871062203	-4,2988761	Agrn	2,380263444
1398482_at	1,251046101	5,576727556	2,062637815	0,048662426	0,871062203	-4,322577767	Bcl3	2,380139448
1393701_at	1,226337981	6,361331212	3,118522165	0,004220026	0,871062203	-3,948883286	Dbr1	2,339723392
1374157_at	1,218970326	7,333225946	2,065188916	0,048403399	0,871062203	-4,321746172	Pde4b	2,327805189
1387459_at	1,217336965	7,860843148	2,129998551	0,042220234	0,871062203	-4,300447183	Pkib	2,325171233
1380583_s_at	1,20431706	6,70407403	2,175691026	0,038294718	0,871062203	-4,28523911	Csf1	2,304281639
1384202_at	1,194106548	9,693378665	2,158211808	0,039756042	0,871062203	-4,2910749	Tesc	2,288030919
1391211_at	1,178100778	6,20029422	2,400621943	0,023354783	0,871062203	-4,208343595	Atp11c	2,262786985
1378634_at	1,175960425	6,713924393	3,407564362	0,00202729	0,871062203	-3,842417505	Hdac8	2,259432449
1390777_at	1,168949082	8,202655986	2,520307311	0,017791014	0,871062203	-4,166267363	Sc5dl	2,248478489
1373416_at	1,167462698	9,471749592	2,302698451	0,029046347	0,871062203	-4,242205691	Fndc3b	2,246163113
1391871_at	1,159536215	7,159140231	2,115626323	0,043527201	0,871062203	-4,305198596	Stard13	2,23385604
1389351_at	1,144804608	7,048078123	2,352969392	0,025983367	0,871062203	-4,224890522	Lrrfip1	2,211161817
1379538_at	1,141713588	7,078519549	2,336112116	0,026976041	0,871062203	-4,230713402	Cenpj	2,206429403
1378592_at	1,132001427	8,584816439	2,12154803	0,042984393	0,871062203	-4,303242802	Trim59	2,1916257
1371171_at	1,11658837	8,63292337	2,154713294	0,040054456	0,871062203	-4,292240283	RT1-EC2	2,168336059
1393085_at	1,083435665	7,847167842	2,508290633	0,018288634	0,871062203	-4,170522694	Mitd1	2,119076491
1368618_at	1,079331406	7,477657814	2,23737425	0,033515147	0,871062203	-4,264473068	Grb14	2,113056592
1388015_at	1,064707454	4,572536585	2,267953986	0,031351101	0,871062203	-4,254083162	Ptprz1	2,091745678
1384177_at	1,05217584	6,66157579	2,35103959	0,026095295	0,871062203	-4,22555795	Gramd3	2,07365493
1389007_at	1,050419377	6,095644772	2,691702341	0,011930505	0,871062203	-4,104939627	Fam189a2	2,071131817
1367593_at	1,045188681	8,484985511	2,246999819	0,032819855	0,871062203	-4,26120924	Sepw1	2,063636231
1370517_at	1,04114195	7,025983949	2,243707468	0,033056195	0,871062203	-4,262326296	Nptx1	2,057855883
1398062_at	1,020453631	6,751716534	2,881768082	0,00756484	0,871062203	-4,035852806	Smap2	2,028556704
1380071_at	1,019873541	8,428923589	2,154333487	0,040086973	0,871062203	-4,292366745	Parp12	2,02774121
1385455_at	1,018822047	7,488223846	2,959792884	0,006252751	0,871062203	-4,007267758	Prpf38b	2,02626385
1382058_at	1,015102152	9,498439684	2,668051039	0,012615179	0,871062203	-4,113466037	Rras2	2,02104599
1385089_at	1,005352738	5,663036597	2,218256799	0,034935604	0,871062203	-4,270937043	Grhl1	2,007434253
1374872_at	1,001553335	6,762582056	3,114794232	0,004259526	0,871062203	-3,950256741	Rasgrp2	2,002154539
1398710_at	-1,004506999	5,696830485	-2,303212586	0,029013428	0,871062203	-4,24202937	Cyp2u1	0,498440431
1383205_at	-1,005306922	8,596559648	-2,19639355	0,036626345	0,871062203	-4,278298888	Dact2	0,49816414
1378632_at	-1,009434721	4,54145432	-2,165869155	0,039109824	0,871062203	-4,288521061	Ppfia4	0,496740843
1373345_at	-1,014515696	9,53211725	-2,161945501	0,039439764	0,871062203	-4,28983019	Amigo2	0,494994467
1382868_at	-1,016608087	5,602274314	-2,18196518	0,037782038	0,871062203	-4,283138998	Sema6a	0,494277079
1377899_at	-1,017435223	6,921735479	-2,119683254	0,043154671	0,871062203	-4,30385898	RGD1304982	0,493993778
1391944_at	-1,071182103	8,47457317	-2,09114424	0,045836671	0,871062203	-4,313255721	Fam184a-ps1	0,475928877
1387476_at	-1,234070448	7,224494546	-2,570786896	0,015834327	0,871062203	-4,148323322	Kcnd2	0,425116321
1379863_at	-1,251567644	7,314776614	-2,432257409	0,021746613	0,871062203	-4,197290906	Kcnd2	0,419991593
1383654_a_at	-1,254684628	6,284159459	-2,277845861	0,030678624	0,871062203	-4,250709325	Fn3k	0,419085169
1387702_at	-1,286644494	5,417845115	-2,394074305	0,023700939	0,871062203	-4,210624587	Galr1	0,409903299
1379824_at	-1,424720775	8,440864474	-2,131131627	0,0421187	0,871062203	-4,300071926	Tox	0,372491452
1370834_at	-1,457628144	6,821901903	-2,122404463	0,04290639	0,871062203	-4,302959721	Hs3st1	0,36409122
1383531_at	-1,565233954	7,314497736	-2,232938473	0,033840011	0,871062203	-4,265975076	Crebrf	0,337922905
1370606_at	-2,054370886	5,987699874	-2,177335528	0,038159741	0,871062203	-4,284688926	P2ry1	0,240753573

Supplementary table 1: List of normalized gene expression profile of the 45 min IL-1β exposed JNK1 knockdown (KD) INS-1 cells, subtracted the gene expression pattern in IL-1β non-exposed JNK1 KD INS-1 cells to gene expression profile of 45 min IL-1β exposed NS INS-1 cells subtracted the gene expression pattern in the IL-1β non-exposed NS INS-1 cells.

ID	logFC	AveExpr	t	P.Value	adj.P.Val	B	Diff JNK2 genes	Fold Change
1375877_at	2,643791349	8,624173612	2,705768019	0,011539958	0,325371531	-2,740685564	Syt4	6,249719104
1384971_at	2,62847873	5,221109076	2,670343861	0,012547233	0,325488805	-2,803814642	Depdc7	6,183736006
1382215_at	2,216028514	5,697247148	2,859134935	0,007991704	0,312106427	-2,462735138	RGD1562997	4,646126756
1386965_at	2,101927461	7,330710513	2,332464564	0,027195328	0,367888461	-3,382830505	Lpl	4,292825297
1391573_at	2,005235661	7,474612044	2,28786338	0,030010944	0,371620236	-3,455816295	Tnfrsf21	4,014542708
1393174_at	1,993177804	6,604301672	2,202521215	0,03614521	0,380176208	-3,592994792	Fam171b	3,98112951
1393584_at	1,965596687	7,547521036	2,092278087	0,045727353	0,393646739	-3,765146623	Tnfrsf21	3,905742092
1377089_a_at	1,788816945	9,475484708	2,736930018	0,010716932	0,319239334	-2,684808828	Tspan5	3,455314298
1373223_at	1,781603929	8,069094044	2,306156207	0,028825601	0,370449471	-3,425986918	Fam171b	3,43808194
1378362_at	1,768362154	5,783758686	3,693764961	0,000963528	0,312106427	-0,852419991	C1ql3	3,406669886
1389199_at	1,759144264	7,164240328	3,829024192	0,000674576	0,312106427	-0,58141666	RGD1309079	3,384972854
1380443_at	1,738687537	7,265679744	2,772463345	0,009845707	0,316103167	-2,620712328	Pwp1	3,337314241
1389666_at	1,682001941	6,611173328	2,574363413	0,015703553	0,335332806	-2,972684713	Rom1	3,208728982
1374245_at	1,668749024	5,731721321	2,098951322	0,045088644	0,392870811	-3,754893882	Nhej1	3,179387859
1379612_at	1,664423737	8,157380902	2,798963537	0,009239756	0,314059443	-2,57265213	Mapk8	3,169870137
1391045_at	1,587841949	6,79394251	2,498761707	0,018692335	0,345082347	-3,103343492	Dgat2	3,005993624
1393612_a_at	1,577492285	4,271547706	2,679048047	0,012292389	0,325371531	-2,788342045	Depdc7	2,98450627
1387459_at	1,57570672	7,860843148	2,757045196	0,010215238	0,316103167	-2,648573469	Pkib	2,980814754
1387818_at	1,567005495	5,319457387	2,093601314	0,045600069	0,393579635	-3,763115376	Casp4	2,962890882
1383257_at	1,565233414	3,877720865	2,356636096	0,025771904	0,364702336	-3,34291653	Rbfox1	2,959253759
1384147_at	1,55574593	8,229598231	2,51554708	0,017986623	0,344696449	-3,074520289	Eif1a	2,939856896
1369105_a_at	1,550658785	8,459135664	2,576556392	0,015623865	0,335332806	-2,968862991	Pkib	2,929508803
1370781_a_at	1,54777685	8,240116555	2,60501675	0,014623143	0,332839955	-2,919106908	Kcnip1	2,923662642
1384882_at	1,544900618	8,151551996	2,539366155	0,017027368	0,34097497	-3,033435209	Hectd2	2,917839682
1383736_at	1,525479	7,36121149	2,731371265	0,010859594	0,321029013	-2,69479942	Elavl2	2,878822822
1390458_at	1,515608273	5,430770545	2,269289456	0,03125954	0,373363202	-3,485952416	LOC100362458	2,859193526
1394316_a_at	1,499877158	8,581619759	2,431947607	0,021761857	0,355220726	-3,216985247	Tspan5	2,828186301
1393220_at	1,488734418	6,261976799	2,645885322	0,013289982	0,325488805	-2,84715391	Armc8	2,806426776
1377163_at	1,480988151	5,220833343	2,460233892	0,020409792	0,348984246	-3,169089262	Inhbb	2,791398603
1383326_a_at	1,476212758	9,288416461	2,922164566	0,006856016	0,312106427	-2,346467688	Pdcd4	2,782174209
1390698_at	1,388185668	7,585271522	2,255355936	0,03222685	0,374779711	-3,508458147	Mapk1ip1l	2,617492981
1392818_at	1,383991316	9,466796717	3,12509542	0,004151229	0,312106427	-1,965011978	Gas5	2,609894186
1394567_at	1,36435125	9,39057544	2,672385716	0,012487012	0,325488805	-2,800187335	Qsox2	2,574605253
1387662_at	1,362862133	5,462116709	2,291248479	0,029788317	0,37099995	-3,450307474	Syt4	2,571949174
1393707_at	1,348396054	8,765720368	2,654088448	0,013036419	0,325488805	-2,832641282	Bcl2l2	2,546288791
1377474_at	1,334275748	7,079784239	2,475221378	0,019725127	0,346650297	-3,143582841	Abhd13	2,52148868
1379314_at	1,327803281	8,907352521	2,379406372	0,02449349	0,362787914	-3,305089757	Ppp3cb	2,51020168
1387116_at	1,32159313	9,517497028	2,540115336	0,016997979	0,340605766	-3,032139496	Dnajb9	2,499419617
1392211_at	1,321551789	7,3802041	3,008605691	0,005544986	0,312106427	-2,185248359	Sec24b	2,499347997
1384217_at	1,303100972	4,967328833	2,622749193	0,014030106	0,330888695	-2,887959421	Zhx2	2,467587035
1382186_a_at	1,287705424	6,753997629	2,232433344	0,033877185	0,377010577	-3,545292039	Gpatch4	2,441394479
1388628_at	1,287241341	9,783203001	2,140862701	0,041255637	0,388299247	-3,690000759	Tmed3	2,440609263
1383768_at	1,285238697	7,276327115	2,413910396	0,022666445	0,357055571	-3,247358736	Elavl2	2,437223738
1397886_at	1,283667022	6,792319495	2,54240298	0,016908528	0,340290174	-3,0281817	Zc3h8	2,434570068
1388928_at	1,275060555	6,087069803	2,77615436	0,009759125	0,316103167	-2,614031452	Cfl2	2,420089756
1387112_at	1,266922799	6,047872504	2,55904783	0,016270689	0,336254768	-2,999326193	Plp1	2,406477274
1384202_at	1,260034291	9,693378665	2,277368707	0,03071076	0,371918382	-3,47286275	Tesc	2,395014334
1372440_at	1,258327442	8,259707802	2,59126836	0,015098886	0,335200918	-2,943179136	Serpine2	2,392182475
1393952_at	1,248824615	6,62938367	2,12323459	0,042830904	0,390676059	-3,717399718	Ccdc68	2,37647729
1373350_at	1,240777757	7,693985858	2,716876896	0,011239998	0,323959878	-2,720802578	Psip1	2,363259013
1377461_at	1,23386559	5,659086047	2,377463132	0,024600287	0,362787914	-3,308326433	LOC100911534	2,351963368
1369886_a_at	1,225524964	5,843883686	2,18752668	0,037332749	0,382777575	-3,616750975	Cabp1	2,338405234
1379567_at	1,221015256	5,421590526	3,137336256	0,004025964	0,312106427	-1,941684705	Med13	2,331107047
1398582_at	1,213178135	5,850485662	2,537809655	0,017088578	0,341980495	-3,036126515	Rps6ka5	2,318478157
1369679_a_at	1,21118577	5,499056454	3,139761784	0,004001576	0,312106427	-1,937058411	Nfia	2,315278543
1388661_at	1,202541755	6,717107755	2,803425177	0,009141252	0,314059443	-2,564539303	Mtfp1	2,301447856
1383327_at	1,202330167	8,808104799	2,81513426	0,008887421	0,312106427	-2,543219242	Pdcd4	2,301110345
1395519_at	1,198662868	6,249956247	2,313674563	0,028350872	0,369397265	-3,413684462	MGC95152	2,295268401
1396233_at	1,195914937	5,652966525	2,137851043	0,041521042	0,388657555	-3,694692387	Arsk	2,290900718
1381144_at	1,194587064	6,929857964	2,62418362	0,013983126	0,33080261	-2,885434951	Impad1	2,288793116
1379027_at	1,191493604	7,620515633	2,549435967	0,016636247	0,339020628	-3,016001875	Wwc1	2,283890691
1387521_at	1,183949513	9,125036282	2,740654943	0,010622319	0,318788805	-2,678108548	Pdcd4	2,27197902
1368053_at	1,181228558	6,607378336	2,57773004	0,015581371	0,335332806	-2,966816945	Pard3	2,267698056
1381895_at	1,179695085	5,955836855	2,301441881	0,029126947	0,370449471	-3,43368846	Nrf1	2,265288949
1380619_at	1,174745174	7,876912251	2,595739037	0,01494263	0,333665403	-2,935358816	RGD1305537	2,257530022
1374400_at	1,16726108	9,120140554	3,167487111	0,003732662	0,312106427	-1,884084661	Efr3a	2,245849232
1385668_at	1,164213556	4,942913142	2,471152763	0,019908869	0,348422021	-3,150515745	LOC683460	2,241110146
1373249_at	1,160532266	8,573630544	2,752822593	0,010318691	0,316307086	-2,656190752	Ubl4a	2,23539885
1398706_at	1,158502694	7,486201775	2,648623622	0,013204833	0,325488805	-2,842312006	LOC100909675	2,232256319
1390272_at	1,158313613	7,145500347	2,359885925	0,025585796	0,364349955	-3,337531173	Dph5	2,231963776
1371038_at	1,157667741	6,055536915	2,168568	0,038884315	0,384284654	-3,646636104	Cebpg	2,230964785
1370517_at	1,157119826	7,025983949	2,493644977	0,018912486	0,345082347	-3,112108209	Nptx1	2,230117656
1377910_at	1,152124688	4,20058224	3,290933118	0,002731839	0,312106427	-1,646272118	Nop58	2,222409526
1373022_at	1,150686109	10,53779405	2,083860199	0,046544505	0,395825555	-3,778048281	Rab1a	2,220194561
1379825_at	1,1418937	9,141009922	2,658805264	0,012892671	0,325488805	-2,824285997	Poc1b	2,206704881
1388015_at	1,138270265	4,572536585	2,424651556	0,022123698	0,355421643	-3,22928722	Ptprz1	2,201169533
1382428_at	1,134681168	6,94372164	2,829391279	0,008587315	0,312106427	-2,517203962	Tasp1	2,195700329
1395455_at	1,13296149	9,033135941	2,824008952	0,008699472	0,312106427	-2,527032454	Hnrnph3	2,193084636

Supplementary table 2

1378081_at	1,127014836	6,355723561	2,942993368	0,006515585	0,312106427	-2,307802423	LOC100912462	2,184063552
1382079_at	1,118904726	6,739792252	2,158909315	0,039696785	0,386031992	-3,661795747	Mapk8	2,171820283
1385158_at	1,112942523	8,32206773	2,495858382	0,018816961	0,345082347	-3,108317991	Chmp1b	2,162863357
1392043_at	1,112009258	6,667052389	2,314645147	0,028290107	0,36935098	-3,412094475	Insr	2,161464675
1389747_at	1,111686572	7,256282164	2,718261455	0,011203129	0,323496839	-2,718321614	Slc26a8	2,160981277
1374598_at	1,106753356	7,703404345	2,168694593	0,038873766	0,384284654	-3,646437116	Mapk8	2,15360454
1398300_at	1,102709231	9,183934433	2,241444103	0,03321956	0,37576696	-3,530841345	Atp1b3	2,147576065
1371094_at	1,100893689	6,812404016	3,048521596	0,005023543	0,312106427	-2,110152002	Lhx2	2,144875174
1398424_at	1,094454748	10,30752563	2,827638959	0,00862368	0,312106427	-2,520404767	Wsb2	2,13532364
1381906_at	1,08995768	7,691931895	2,261457705	0,031799963	0,374779711	-3,498613185	Tpd52	2,128677921
1393251_at	1,089500433	9,503481764	2,219784764	0,034820111	0,379026195	-3,565513967	Arl10	2,128003366
1393613_at	1,085133396	8,246782226	2,876532572	0,007661623	0,312106427	-2,430755489	Zfp462	2,12157164
1383396_at	1,082773063	9,436116572	2,352074242	0,026035231	0,365693151	-3,35046851	Fndc3a	2,118103464
1395799_at	1,072107313	6,629097754	3,772752974	0,000782696	0,312106427	-0,694362784	Itsn2	2,102502205
1385009_at	1,071263653	6,156501992	2,080203587	0,046903485	0,396157383	-3,783641572	Nol8	2,101273062
1392953_at	1,06851983	7,442469159	2,165844815	0,039111863	0,384999329	-3,650914741	Ptpla	2,097280505
1385333_at	1,068500939	7,101584932	2,139388162	0,041385392	0,388577411	-3,692298378	Aatk	2,097253043
1383489_at	1,066468294	7,223051656	2,13618656	0,04166838	0,388657555	-3,697283474	Il6st	2,094300256
1389918_at	1,066380677	8,591880517	3,168325387	0,003724807	0,312106427	-1,882480386	LOC100360205	2,094173071
1382161_at	1,066122361	7,729757955	2,890019332	0,007414651	0,312106427	-2,405904888	Mphosph10	2,09379814
1382059_at	1,063295579	7,442062026	2,544762561	0,016816721	0,340017919	-3,024097401	Fbxo30	2,089699619
1391432_at	1,062540154	8,513508722	2,854524304	0,008081405	0,312106427	-2,471195505	Wdr75	2,088605695
1394435_at	1,057987672	8,564935315	2,222694336	0,034601139	0,379026195	-3,560868805	Vangl1	2,082025404
1385639_at	1,05685415	8,333683541	3,016005902	0,005444576	0,312106427	-2,171355965	Casp8ap2	2,080390205
1372133_at	1,05222829	10,2561388	2,415413626	0,02258976	0,357055571	-3,244832446	Rras2	2,07373032
1396315_at	1,046396573	7,190995457	3,140494456	0,003994237	0,312106427	-1,935660701	Ccdc112	2,065364728
1383403_at	1,046391213	9,287921134	3,543566597	0,001426429	0,312106427	-1,151094649	LOC678772	2,065357054
1377704_at	1,046241358	6,657752627	2,793578247	0,009359982	0,315028211	-2,582436354	Msl2	2,065142534
1374932_at	1,041200572	9,201785263	2,1273444	0,042458926	0,389884012	-3,711025443	LOC100361444	2,057939502
1376867_at	1,03913212	7,538660014	3,270318456	0,002878777	0,312106427	-1,686196058	Lgalsl	2,054991064
1374541_at	1,034617441	8,108229866	2,266143344	0,031475628	0,374313973	-3,491041714	Gys1	2,048570355
1386910_a_at	1,032123589	8,693441915	2,102842101	0,044719921	0,392311659	-3,748905923	Apex1	2,045032242
1374591_at	1,031897951	5,446892124	2,130668961	0,042160132	0,389000054	-3,705863061	Ptprd	2,044712424
1372189_at	1,031361343	8,324955672	2,299862425	0,029228545	0,370449471	-3,436266552	Dnajc13	2,043952037
1391757_at	1,030895735	5,071105347	3,048426621	0,005024726	0,312106427	-2,110331153	Ptpn4	2,043292489
1389228_at	1,030765141	9,10210877	2,228037636	0,034202234	0,378112289	-3,552328018	Fam136a	2,043107537
1382210_at	1,025233769	5,832031642	3,295518622	0,002700151	0,312106427	-1,637380542	Rap2c	2,035289148
1397960_at	1,023478866	8,257789327	2,967472884	0,006135975	0,312106427	-2,262210585	Armcx3	2,032814916
1375846_at	1,02100461	7,66302997	2,333074268	0,027158561	0,367888461	-3,381826791	Xpr1	2,029331577
1367930_at	1,017835718	9,635420002	2,311595533	0,028481432	0,369866369	-3,417088894	Gap43	2,024879026
1378936_at	1,017497478	8,162094214	3,251099635	0,003022593	0,312106427	-1,723342985	Cstf2	2,024404347
1374793_at	1,0162056	8,204496023	2,636722274	0,013578647	0,327097086	-2,863337246	Wdr3	2,022592383
1381206_at	1,014518283	4,080408668	2,054738398	0,049472299	0,39921783	-3,822407904	Plcxd2	2,020228225
1383328_x_at	1,013878284	9,546021508	2,291486971	0,029772689	0,37099995	-3,449919167	Pdcd4	2,019332223
1368983_at	1,01252335	7,771746472	2,341167866	0,026674757	0,366693331	-3,368487697	Hbegf	2,017436619
1376174_at	1,011134526	5,370362158	2,38868721	0,023989253	0,360873145	-3,28960979	Serpina11	2,015495449
1383336_at	1,009706177	8,220510989	3,001825036	0,005638533	0,312106427	-2,197965452	Pnn	2,013500983
1378930_a_at	1,006671209	6,942302175	2,250716184	0,032554911	0,374779711	-3,51593295	LOC100912518	2,009269675
1370773_a_at	-1,003043006	4,721161232	-2,961639959	0,006224474	0,312106427	-2,273088604	Kcnip2	0,498946486
1398289_a_at	-1,047125872	7,028917451	-2,508401911	0,018283967	0,344696449	-3,086802892	Crhr1	0,48393129
1376224_at	-1,060083784	6,47499032	-2,608883285	0,014491879	0,332362059	-2,91232476	LOC100910536	0,479604206
1368146_at	-1,0663904	10,58901567	-3,196270512	0,003471843	0,312106427	-1,828913525	Dusp1	0,477512234
1386998_at	-1,069165686	7,042623269	-2,074219219	0,04749629	0,397386901	-3,792781053	Aldoc	0,476594535
1386754_at	-1,092818705	8,123749323	-2,192971911	0,036897504	0,381410234	-3,608136156	Trim14	0,468844461
1374404_at	-1,114599667	6,013390843	-2,275078507	0,030865428	0,372213976	-3,476576197	Jun	0,461819288
1373161_at	-1,156092528	10,92635004	-3,080460006	0,004640319	0,312106427	-2,049783107	Tmem98	0,448726245
1387776_at	-1,178042376	7,296537955	-2,084208678	0,046510421	0,395825555	-3,777514886	Tgm2	0,441950784
1367892_at	-1,212612557	7,968016226	-2,883200471	0,007538563	0,312106427	-2,418475748	Pdk2	0,431486535
1369788_s_at	-1,277282655	7,311762809	-2,411091373	0,022810897	0,357287146	-3,252093823	Jun	0,412571865
1373043_at	-1,345114276	9,746506739	-3,055506362	0,004937239	0,312106427	-2,096970658	Sdf2l1	0,393622808
1387870_at	-1,422137228	8,54191257	-2,889375821	0,007426262	0,312106427	-2,407091791	Zfp36	0,3731591
1368147_at	-1,473169265	7,182111156	-3,549174623	0,00140579	0,312106427	-1,139993026	Dusp1	0,360190175
1370478_at	-1,704752864	8,826179152	-2,391041833	0,023862843	0,360073046	-3,285676701	Myo16	0,306773791
1379363_at	-1,83736439	6,511229004	-2,101822667	0,044816271	0,392603159	-3,750475568	LOC679818	0,279832534
1370490_at	-1,903431257	6,25855324	-2,326011407	0,027587262	0,368406744	-3,393444088	Pcdhb12	0,267306855
1370140_a_at	-2,243289604	4,905093357	-2,671735574	0,012506157	0,325488805	-2,80134245	Pax4	0,211204195
1387981_at	-2,350792289	6,285091041	-2,450939043	0,020845316	0,350709323	-3,184863073	Olr59	0,196038336

Supplementary table 2: List of normalized gene expression profile of the 45 min IL-1β exposed JNK2 KD INS-1 cells, subtracted the gene expression pattern in IL-1β non-exposed JNK2 KD INS-1 cells to gene expression profile of 45 min IL-1β exposed NS INS-1 cells subtracted the gene expression pattern in the IL-1β non-exposed NS INS-1 cells.

ID	logFC	AveExpr	t	P.Value	adj.P.Val	B	Diff JNK3 genes	Fold Change
1375877_at	2,49432843	8,624173612	2,552801338	0,016507399	0,386447016	-3,009933834	Syt4	5,634659492
1384971_at	2,297018213	5,221109076	2,333604002	0,027126652	0,408715214	-3,382082644	Depdc7	4,914409963
1386965_at	2,156659677	7,330710513	2,39319975	0,023747528	0,397697565	-3,282838606	Lpl	4,458812945
1391573_at	2,065221679	7,474612044	2,35630412	0,025790985	0,405108087	-3,344458413	Tnfrsf21	4,184982758
1393584_at	2,048859735	7,547521036	2,180907384	0,03786804	0,423317957	-3,629217296	Tnfrsf21	4,137788014
1389199_at	1,970289668	7,164240328	4,288611774	0,000197454	0,33056701	0,362013392	RGD1309079	3,91846787
1380443_at	1,965935452	7,265679744	3,134826622	0,004051348	0,35703421	-1,942128939	Pwp1	3,906659322
1382215_at	1,91120239	5,697247148	2,465846214	0,020150886	0,393279383	-3,159867704	RGD1562997	3,761224422
1387459_at	1,85774592	7,860843148	3,250534758	0,003026923	0,35703421	-1,719195807	Pkib	3,624409383
1369105_a_at	1,837250262	8,459135664	3,052753419	0,004971085	0,35703421	-2,098446793	Pkib	3,573283208
1384882_at	1,783539083	8,151551996	2,931618209	0,006699466	0,38019467	-2,326104506	Hectd2	3,442696693
1390698_at	1,751416282	7,585271522	2,845489043	0,008259951	0,380993456	-2,485544407	Mapk1ip1l	3,366889288
1391544_at	1,70961008	6,576724828	2,425338781	0,022089382	0,395257589	-3,228700623	Pnpla3	3,27072413
1378362_at	1,649837148	5,783758686	3,446189253	0,001835384	0,35703421	-1,336347701	C1ql3	3,137982155
1373223_at	1,633074613	8,069094044	2,113895852	0,043686971	0,43547049	-3,734237538	Fam171b	3,101733236
1383326_a_at	1,632839568	9,288416461	3,232207486	0,003170683	0,35703421	-1,754693415	Pdcd4	3,101227941
1370517_at	1,624570333	7,025983949	3,501021726	0,001592879	0,35703421	-1,227912354	Nptx1	3,083503177
1384147_at	1,614245725	8,229598231	2,61013771	0,014449529	0,386447016	-2,909516517	Eif1a	3,061514943
1370234_at	1,585816026	11,14440306	2,205143934	0,03594102	0,42025391	-3,590704698	Fn1	3,001775381
1379482_at	1,584842709	5,838486177	2,085715788	0,046363271	0,439384818	-3,777744634	Tm6sf1	2,999750909
1392818_at	1,523754811	9,466796717	3,440685736	0,001861612	0,35703421	-1,34720598	Gas5	2,875384347
1377089_a_at	1,501426046	9,475484708	2,2972155	0,02939953	0,415762301	-3,44192828	Tspan5	2,831224293
1390458_at	1,459379606	5,430770545	2,185099416	0,037528244	0,422332804	-3,622575863	LOC100362458	2,749900857
1370781_a_at	1,452169019	8,240116555	2,44410208	0,021171107	0,394076281	-3,196898805	Kcnip1	2,736191147
1379612_at	1,424002224	8,157380902	2,394660814	0,023669742	0,397421884	-3,280386713	Mapk8	2,683288586
1393952_at	1,419064234	6,62938367	2,412673671	0,022729714	0,396822987	-3,250085666	Ccdc68	2,67412005
1393707_at	1,418314251	8,765720368	2,791710535	0,009402021	0,383370555	-2,583992214	Bcl2l2	2,672730274
1384742_at	1,411907315	5,703594938	3,520026645	0,001516318	0,35703421	-1,190225502	Atrx	2,66088713
1374400_at	1,410836119	9,120140554	3,82845389	0,000675594	0,35703421	-0,572485497	Efr3a	2,658912164
1372440_at	1,403807024	8,259707802	2,890853846	0,007399619	0,380993456	-2,401830339	Serpine2	2,645988927
1387662_at	1,40347539	5,462116709	2,359527626	0,025606255	0,404682083	-3,339097737	Syt4	2,64538076
1393220_at	1,398224076	6,261976799	2,485023866	0,019288832	0,393279383	-3,12705176	Armc8	2,635769252
1390777_at	1,383126412	8,202655986	2,982083362	0,005919513	0,371192657	-2,231726984	Sc5dl	2,608330008
1388628_at	1,381061431	9,783203001	2,296898656	0,029420058	0,415762301	-3,442446818	Tmed3	2,604599283
1379027_at	1,375624384	7,620515633	2,943420149	0,00650878	0,378175195	-2,304094632	Wwc1	2,594801871
1373350_at	1,373359994	7,693985858	3,007186433	0,005564443	0,371110392	-2,1845295	Psip1	2,59073238
1379314_at	1,361471707	8,907352521	2,439739757	0,021381416	0,394076281	-3,204305188	Ppp3cb	2,5694716
1383736_at	1,354818727	7,36121149	2,42580392	0,022066183	0,395069793	-3,227913988	Elavl2	2,557649776
1384202_at	1,351580032	9,693378665	2,442827225	0,021232371	0,394076281	-3,199064056	Tesc	2,551914568
1390272_at	1,348092838	7,145500347	2,746531922	0,010474635	0,385404569	-2,666004932	Dph5	2,545753685
1398217_at	1,336959602	6,065479288	2,983186878	0,005903462	0,371192657	-2,229655659	Zbtb41	2,526183785
1393612_a_at	1,324792142	4,271547706	2,249888532	0,032613747	0,416147597	-3,518884998	Depdc7	2,504967945
1395455_at	1,322012433	9,033135941	3,295235522	0,002702097	0,35703421	-1,632338513	Hnrnph3	2,500146152
1393613_at	1,310620779	8,246782226	3,474267197	0,001707047	0,35703421	-1,280877891	Zfp462	2,4804825
1385916_at	1,307749205	4,171838432	3,59593043	0,001244653	0,35703421	-1,039215283	LOC686240	2,475550201
1367930_at	1,278581977	9,635420002	2,903773501	0,007170548	0,380993456	-2,377880646	Gap43	2,42600408
1385639_at	1,27645289	8,333683541	3,642687543	0,001101543	0,35703421	-0,945827477	Casp8ap2	2,422426495
1383336_at	1,266173459	8,220510989	3,764294284	0,000800351	0,35703421	-0,701805251	Pnn	2,405227667
1388928_at	1,260401641	6,087069803	2,744237909	0,010532051	0,385404569	-2,670151925	Cfl2	2,395624249
1383023_at	1,249620352	5,123981602	2,39605195	0,023595895	0,397107506	-3,278051345	Ube2h	2,377788428
1373022_at	1,244714025	10,53779405	2,254142112	0,032312385	0,416147597	-3,512009701	Rab1a	2,369715763
1396315_at	1,243422373	7,190995457	3,731817523	0,000871826	0,35703421	-0,767122918	Ccdc112	2,367595095
1382079_at	1,234214974	6,739792252	2,381398652	0,024384438	0,401002921	-3,302610041	Mapk8	2,352533023
1391022_at	1,231525242	8,278680222	2,744440559	0,010526967	0,385404569	-2,669785653	Lamb3	2,348151093
1387112_at	1,222354316	6,047872504	2,469024287	0,02000562	0,393279383	-3,154439555	Plp1	2,333271705
1381206_at	1,220041122	4,080408668	2,470990798	0,019916216	0,393279383	-3,151078753	Plcxd2	2,329533573
1378936_at	1,203341518	8,162094214	3,844906996	0,000646802	0,35703421	-0,539267175	Cstf2	2,302724024
1373249_at	1,19588721	8,573630544	2,836685739	0,008437488	0,380993456	-2,501719638	Ubl4a	2,290856689
1382161_at	1,195637098	7,729757955	3,241104825	0,003100091	0,35703421	-1,737468832	Mphosph10	2,290459572
1372133_at	1,184700879	10,2561388	2,719507423	0,011170046	0,385404569	-2,714749929	Rras2	2,273162591
1377474_at	1,182195643	7,079784239	2,193096842	0,036887572	0,421363014	-3,609882486	Abhd13	2,269218677
1392211_at	1,177644937	7,3802041	2,68099161	0,012236148	0,385404569	-2,783805725	Sec24b	2,262072136
1383768_at	1,167534491	7,276327115	2,192840638	0,036907943	0,421363014	-3,610289597	Elavl2	2,246274891
1393684_at	1,165566751	8,312076574	2,493777692	0,018906746	0,393279383	-3,112024653	Hells	2,243213212
1382058_at	1,163970812	9,498439684	3,059331053	0,004890583	0,35703421	-2,085978077	Rras2	2,240733095
1385061_at	1,158772865	7,961771861	2,846785103	0,008234113	0,380993456	-2,483161076	Tmem229a	2,232674389
1394029_at	1,158075924	7,836722589	2,559630044	0,016248787	0,386447016	-2,998037573	Vma21	2,231596083
1369886_a_at	1,151105294	5,843883686	2,054689719	0,049477327	0,442806641	-3,82518536	Cabp1	2,220839748
1374932_at	1,151056157	9,201785263	2,351797469	0,026051286	0,406453982	-3,351945524	LOC100361444	2,220764109
1377503_at	1,147320032	7,463773477	3,494634042	0,001619445	0,35703421	-1,24056751	Riok2	2,215020471
1378081_at	1,146583697	6,355723561	2,994093872	0,005747025	0,371110392	-2,209165849	LOC100912462	2,213890238
1392449_at	1,144142774	9,926738638	5,103885681	2,16E-05	0,27773779	2,013265162	Rad18	2,210147682
1398706_at	1,14196788	7,486201775	2,610820948	0,014426511	0,386447016	-2,908312722	LOC100909675	2,206818347
1388661_at	1,137548529	6,717107755	2,651909733	0,013103321	0,385404569	-2,835614787	Mtfp1	2,20006863
1394567_at	1,133798565	9,39057544	2,220796946	0,034743794	0,417971197	-3,565685269	Qsox2	2,194357468
1389362_at	1,131242534	7,334257882	2,167228701	0,038996078	0,426622475	-3,65083031	Ptpn3	2,190473154
1398424_at	1,125851655	10,30752563	2,908756172	0,007084009	0,380993456	-2,368631464	Wsb2	2,182303348
1382428_at	1,119409442	6,94372164	2,791310373	0,009411051	0,383370555	-2,584721442	Tasp1	2,172580212

Supplementary table 3

1387116_at	1,118546975	9,517497028	2,149858577	0,04047187	0,428690284	-3,678146893	Dnajb9	2,171281796
1389228_at	1,116542985	9,10210877	2,413449675	0,022689996	0,396822987	-3,248777273	Fam136a	2,168267849
1393892_at	1,114843395	7,550520391	2,443891335	0,021181224	0,394076281	-3,197256785	RGD1306625	2,165714989
1389918_at	1,105198184	8,591880517	3,283656145	0,002782864	0,35703421	-1,654875245	LOC100360205	2,151284284
1383396_at	1,105147705	9,436116572	2,40067798	0,02335184	0,397107506	-3,270279626	Fndc3a	2,151209013
1379825_at	1,104701659	9,141009922	2,572206663	0,015782293	0,386447016	-2,976082662	Poc1b	2,150544014
1387812_at	1,103343421	8,03025528	2,293671382	0,029629892	0,415762301	-3,447725942	Pcsk6	2,148520318
1381144_at	1,094699004	6,929857964	2,404756658	0,023138582	0,397107506	-3,263420102	Impad1	2,135685192
1398300_at	1,093965094	9,183934433	2,223670157	0,034527978	0,417971197	-3,561080422	Atp1b3	2,134599029
1380619_at	1,093466822	7,876912251	2,416144862	0,022552543	0,396822987	-3,244231092	RGD1305537	2,133861917
1383327_at	1,091205261	8,808104799	2,554946554	0,016425749	0,386447016	-3,006198533	Pdcd4	2,130519507
1371094_at	1,086806462	6,812404016	3,009512184	0,005532592	0,371110392	-2,180148509	Lhx2	2,124033414
1381906_at	1,085843077	7,691931895	2,252920676	0,032398664	0,416147597	-3,513984818	Tpd52	2,122615527
1376737_at	1,079474825	10,42141522	2,757023256	0,010215773	0,385404569	-2,647017725	LOC100912041	2,113266662
1378634_at	1,074908899	6,713924393	3,114748742	0,00426001	0,35703421	-1,980515082	Hdac8	2,106589044
1377836_at	1,066235324	6,521445635	3,295675207	0,002699075	0,35703421	-1,631482261	Zfp280d	2,093962091
1393350_at	1,061815377	5,117404053	2,382601246	0,024318826	0,401002921	-3,300597893	LOC100911349	2,087556691
1391432_at	1,056186556	8,513508722	2,837455301	0,008421826	0,380993456	-2,500306562	Wdr75	2,079427745
1398841_at	1,053550949	12,34361563	2,285313821	0,030179614	0,415762301	-3,461375619	Rab1a	2,075632382
1374111_at	1,051845042	8,171467725	3,602151808	0,001224616	0,35703421	-1,026804694	Pnn	2,073179512
1393251_at	1,049051922	9,503481764	2,13737361	0,041563256	0,432733747	-3,697691021	Arl10	2,06916963
1374425_at	1,047573205	7,467267663	2,47697193	0,019646551	0,393279383	-3,14084751	Tle1	2,067049883
1368053_at	1,047155234	6,607378336	2,285149208	0,030190534	0,415762301	-3,461644155	Pard3	2,066451114
1386910_a_at	1,045322231	8,693441915	2,12973293	0,042244068	0,434107442	-3,709614459	Apex1	2,06382727
1395696_at	1,043719437	6,296471949	2,510163907	0,018210224	0,392844707	-3,083815571	Yae1d1	2,061535689
1398599_at	1,043087786	7,990591525	2,155248344	0,040008688	0,426961671	-3,669686378	Stt3a	2,06063329
1373416_at	1,042904645	9,471749592	2,057020679	0,049237096	0,442806641	-3,821638119	Fndc3b	2,060371723
1387521_at	1,042667907	9,125036282	2,41361048	0,022681774	0,396822987	-3,248506114	Pdcd4	2,060033655
1397886_at	1,039324352	6,792319495	2,058463203	0,049088946	0,442806641	-3,819441508	Zc3h8	2,055264899
1374879_x_at	1,038940346	9,125003757	2,921195774	0,006872253	0,380993456	-2,345509862	Larp4b	2,054717916
1378930_a_at	1,030550062	6,942302175	2,304104539	0,028956397	0,415762301	-3,430642921	LOC100912518	2,04280297
1374793_at	1,026665678	8,204496023	2,663862768	0,012740183	0,385404569	-2,814355701	Wdr3	2,037310224
1388015_at	1,025893872	4,572536585	2,185276422	0,037513957	0,422332804	-3,622295249	Ptprz1	2,036220604
1374389_at	1,024215173	8,21670729	2,843129618	0,008307185	0,380993456	-2,489881888	Gucy1b3	2,033852666
1386982_at	1,023049356	8,390237487	3,883762466	0,000583497	0,35703421	-0,460736951	Mgat2	2,032209808
1390507_at	1,019194685	10,21424128	3,113370581	0,004274704	0,35703421	-1,983146552	Isg20	2,026787287
1376719_at	1,016690412	6,350546183	2,512609867	0,01810831	0,392618461	-3,079595934	Tmem38b	2,023272181
1393753_at	1,012721026	7,650165503	2,943400504	0,006509093	0,378175195	-2,304131299	Nudcd1	2,017713065
1376704_a_at	1,009729748	8,581853644	2,630428697	0,013780287	0,386447016	-2,873695119	Ndnl2	2,013533881
1384997_at	1,005670494	7,520168601	2,229639105	0,034083486	0,417615038	-3,551501884	Aen	2,007876443
1374249_at	1,00529757	7,273520667	2,46379238	0,02024528	0,393279383	-3,163373524	Syne4	2,007357491
1393780_at	1,004125033	7,834594587	3,336596121	0,002431642	0,35703421	-1,551634674	Zfp131	2,005726693
1381205_at	1,003945536	6,348050642	2,443778814	0,021186627	0,394076281	-3,197447911	Snapc5	2,005477161
1389747_at	1,00249801	7,256282164	2,451276975	0,020829333	0,394062166	-3,184700575	Slc26a8	2,003465976
1376893_at	-1,029523018	10,35671926	-3,26765548	0,002898304	0,35703421	-1,685974713	Nrsn1	0,489872083
1375788_at	-1,032692571	6,03374311	-2,90436478	0,007160226	0,380993456	-2,376783438	Rpl7	0,488797032
1398289_a_at	-1,050972675	7,028917451	-2,51761697	0,017901324	0,39248496	-3,070950796	Crhr1	0,482642654
1372858_at	-1,083928765	4,69563409	-2,218815952	0,0348933	0,417971197	-3,568857946	LOC100911740	0,471742418
1379863_at	-1,091019009	7,314776614	-2,120252214	0,043102654	0,435210858	-3,724369628	Kcnd2	0,469429688
1386754_at	-1,140537457	8,123749323	-2,288729681	0,029953827	0,415762301	-3,455800546	Trim14	0,453590567
1367892_at	-1,180144502	7,968016226	-2,806001937	0,009084814	0,383370555	-2,557916054	Pdk2	0,441307294
1387476_at	-1,189058234	7,224494546	-2,477018496	0,019644465	0,393279383	-3,140767799	Kcnd2	0,438589071
1387776_at	-1,19996546	7,296537955	-2,122995297	0,042852651	0,435082639	-3,720105003	Tgm2	0,435285703
1373161_at	-1,210269784	10,92635004	-3,224817717	0,003230486	0,35703421	-1,768987234	Tmem98	0,432187789
1389474_at	-1,243447234	9,114820971	-2,160232824	0,039584563	0,426622475	-3,661849599	Mylip	0,42236224
1373108_at	-1,344391652	8,295129885	-3,099316681	0,004427334	0,35703421	-2,009956009	Ppp1r3c	0,393820017
1387981_at	-2,179691141	6,285091041	-2,272548768	0,031037093	0,416147597	-3,48216351	Olr59	0,220722997

Supplementary table 3: List of normalized gene expression profile of the 45 min IL-1β exposed JNK1KD INS-1 cells, subtracted the gene expression pattern in IL-1β non-exposed JNK1 KD INS-1 cells to gene expression profile of 45 min IL-1β exposed NS INS-1 cells subtracted the gene expression pattern in the IL-1β non-exposed NS INS-1 cells.
